# Caffeic acid phenethyl ester induced cell cycle arrest and growth inhibition in androgen-independent prostate cancer cells via regulation of Skp2, p53, p21^Cip1^ and p27^Kip1^

**DOI:** 10.18632/oncotarget.3246

**Published:** 2015-02-16

**Authors:** Hui-Ping Lin, Ching-Yu Lin, Chieh Huo, Ping-Hsuan Hsiao, Liang-Cheng Su, Shih Sheng Jiang, Tzu-Min Chan, Chung-Ho Chang, Li-Tzong Chen, Hsing-Jien Kung, Horng-Dar Wang, Chih-Pin Chuu

**Affiliations:** ^1^ National Institute of Cancer Research, National Health Research Institutes, Miaoli, Taiwan, ROC; ^2^ Institute of Cellular and System Medicine, National Health Research Institutes, Miaoli, Taiwan, ROC; ^3^ Department of Life Sciences, National Central University, Taoyuan, Taiwan, ROC; ^4^ Institute of Biotechnology, National Tsing Hua University, Hsinchu City, Taiwan, ROC; ^5^ Department of Medical Education and Research, China Medical University Beigan Hospital, Yunlin, Taiwan, ROC; ^6^ Department of Medical Education and Research, China Medical University-An Nan Hospital, Tainan, Taiwan, ROC; ^7^ Institute of Molecular and Genomic Medicine, National Health Research Institutes, Miaoli County, Taiwan, ROC; ^8^ Graduate Institute of Basic Medical Science, China Medical University, Taichung, Taiwan, ROC; ^9^ Graduate Program for Aging, China Medical University, Taichung, Taiwan, ROC; ^10^ Biotechnology Center, National Chung Hsing University, Taichung, Taiwan, ROC; ^11^ Ph.D. Program in Environmental and Occupational Medicine, Kaohsiung Medical University, Kaohsiung City, Taiwan, ROC

**Keywords:** Prostate cancer, caffeic acid phenethyl ester, cell cycle arrest, Skp2, p53

## Abstract

Prostate cancer (PCa) patients receiving the androgen ablation therapy ultimately develop recurrent castration-resistant prostate cancer (CRPC) within 1–3 years. Treatment with caffeic acid phenethyl ester (CAPE) suppressed cell survival and proliferation via induction of G1 or G2/M cell cycle arrest in LNCaP 104-R1, DU-145, 22Rv1, and C4–2 CRPC cells. CAPE treatment also inhibited soft agar colony formation and retarded nude mice xenograft growth of LNCaP 104-R1 cells. We identified that CAPE treatment significantly reduced protein abundance of Skp2, Cdk2, Cdk4, Cdk7, Rb, phospho-Rb S807/811, cyclin A, cyclin D1, cyclin H, E2F1, c-Myc, SGK, phospho-p70S6kinase T421/S424, phospho-mTOR Ser2481, phospho-GSK3α Ser21, but induced p21^Cip1^, p27^Kip1^, ATF4, cyclin E, p53, TRIB3, phospho-p53 (Ser6, Ser33, Ser46, Ser392), phospho-p38 MAPK Thr180/Tyr182, Chk1, Chk2, phospho-ATM S1981, phospho-ATR S428, and phospho-p90RSK Ser380. CAPE treatment decreased Skp2 and Akt1 protein expression in LNCaP 104-R1 tumors as compared to control group. Overexpression of Skp2, or siRNA knockdown of p21^Cip1^, p27^Kip1^, or p53 blocked suppressive effect of CAPE treatment. Co-treatment of CAPE with PI3K inhibitor LY294002 or Bcl-2 inhibitor ABT737 showed synergistic suppressive effects. Our finding suggested that CAPE treatment induced cell cycle arrest and growth inhibition in CRPC cells via regulation of Skp2, p53, p21^Cip1^, and p27^Kip1^.

## INTRODUCTION

Prostate cancer is the second most frequently diagnosed cancer of men and the fifth most common cancer overall in the world. Incidence of prostate cancer (PCa) is increasing steadily in almost all countries [1]. According to the statistics of Surveillance Epidemiology and End Results (SEER) of National Cancer Institute, more than 240,000 men were diagnosed with and more than 28,000 men died of cancer of the prostate in 2012 in United States. While surgery is often successful for organ-confined PCa, androgen ablation therapy is the primary treatment for metastatic PCa. However, most PCa patients receiving the androgen ablation therapy will ultimately develop castration-resistant prostate cancer (CRPC) within 1–3 years with a median overall survival time of 1–2 years after relapse [2, 3]. Currently, there is no effective standard therapy for CRPC. Although chemotherapy is usually applied for treatment of CRPC [4], these drugs show little effect on prolonging survival [4]. Undesired side effects of these chemotherapeutic agents include toxic deaths, strokes, thrombosis, neutropenia, edema, dyspnea, malaise, and fatigue [4]. Alternative therapies are therefore in need for CRPC.

Androgen receptor (AR), an androgen-activated transcription factor, belongs to the nuclear receptor superfamily. AR plays essential roles in the development of male sex organs and prostate tissues, maturation of bones, and normal female fertility. AR signaling is important for the development, progression, and metastasis of PCa [5]. Increase in AR mRNA and protein was observed in CRPC tumors compared to the primary prostate tumors [6–11]. LNCaP is a commonly used cell line established from a human lymph node metastatic lesion of prostatic adenocarcinoma [12], which expresses AR and prostate specific antigen (PSA). We have established LNCaP sublines mimic the progression of PCa. An androgen-dependent clonal subline of the LNCaP human prostate cancer cell line called LNCaP 104-S was subjected to long-term androgen deprivation in order to model changes which occur in the PCa cells in patient undergoing androgen-ablation therapy. LNCaP 104-S cells first underwent a G1 cell cycle arrest and subsequently died [13, 14]. However, a small portion of the cells survived and re-started to proliferate after about 40 passages (~half year) in androgen-depleted medium. The surviving LNCaP 104-S cells gave rise to LNCaP 104-R1 cells [13, 14]. Proliferation of LNCaP 104-R1 cells is androgen-independent but is repressed by physiological concentration of androgens [13, 14]. During the transition of LNCaP 104-S cells to LNCaP 104-R1, AR mRNA and protein level increased dramatically. AR transcriptional activity also increased by 20-fold during the progression [13, 14]. Our LNCaP prostate cancer progression model mimics the clinical situations in which AR-positive prostate tumors recur following androgen deprivation [2, 15, 16].

Caffeic acid phenethyl ester (CAPE) is a main bioactive component extracted from honeybee hive propolis. CAPE is a well known NF-κB inhibitor at concentrations of 50 μM to 80 μM by preventing the translocation of p65 unit of NF-κB and the binding between NF-κB and DNA [17]. We previously reported that CAPE dosage dependently suppressed the proliferation of androgen-dependent LNCaP 104-S and AR-negative PC-3 cells [18, 19]. Administration of CAPE by gavage significantly inhibited the tumor growth of LNCaP and PC-3 xenografts in nude mice [18–20]. We discovered that CAPE treatment inhibited cell growth and induced G1 cell cycle arrest by suppressing c-Myc and Akt-related protein signaling networks in LNCaP 104-S and PC-3 cells [18–20]. However, the protein expression profile and response to treatment of chemotherapy drugs or kinase inhibitors was quite different between LNCaP 104-R1 and LNCaP 104-S cells [21]. We therefore used LNCaP 104-R1 cells as well as other CRPC cell lines 22Rv1, DU-145, and LNCaP C4–2 to determine the molecular mechanisms lying underneath of the anticancer effects of CAPE on CRPC cells. Micro-Western Array (MWA) is an antibody-based modified reverse phase array allows detecting protein expression level or phosphorylation status change of 96–384 different antibodies in 6–15 samples simultaneously [22]. We used MWA to determine the changes of signaling protein profile in LNCaP 104-R1 cells being treated with CAPE. Our study suggested that CAPE treatment can efficiently induced G1 or G2/M cell cycle arrest, cellular and growth inhibition in CRPC cells via inhibition of Skp2 as well as induction of p21^Cip1^, p27^Kip1^, and p53 in CRPC cell lines. Our finding implied that CAPE treatment might be a potential therapy for patients with CRPC.

## RESULTS

### CAPE treatment suppressed the proliferation and survival of castration-resistant prostate cancer (CRPC) cell lines

**Figure 1:**
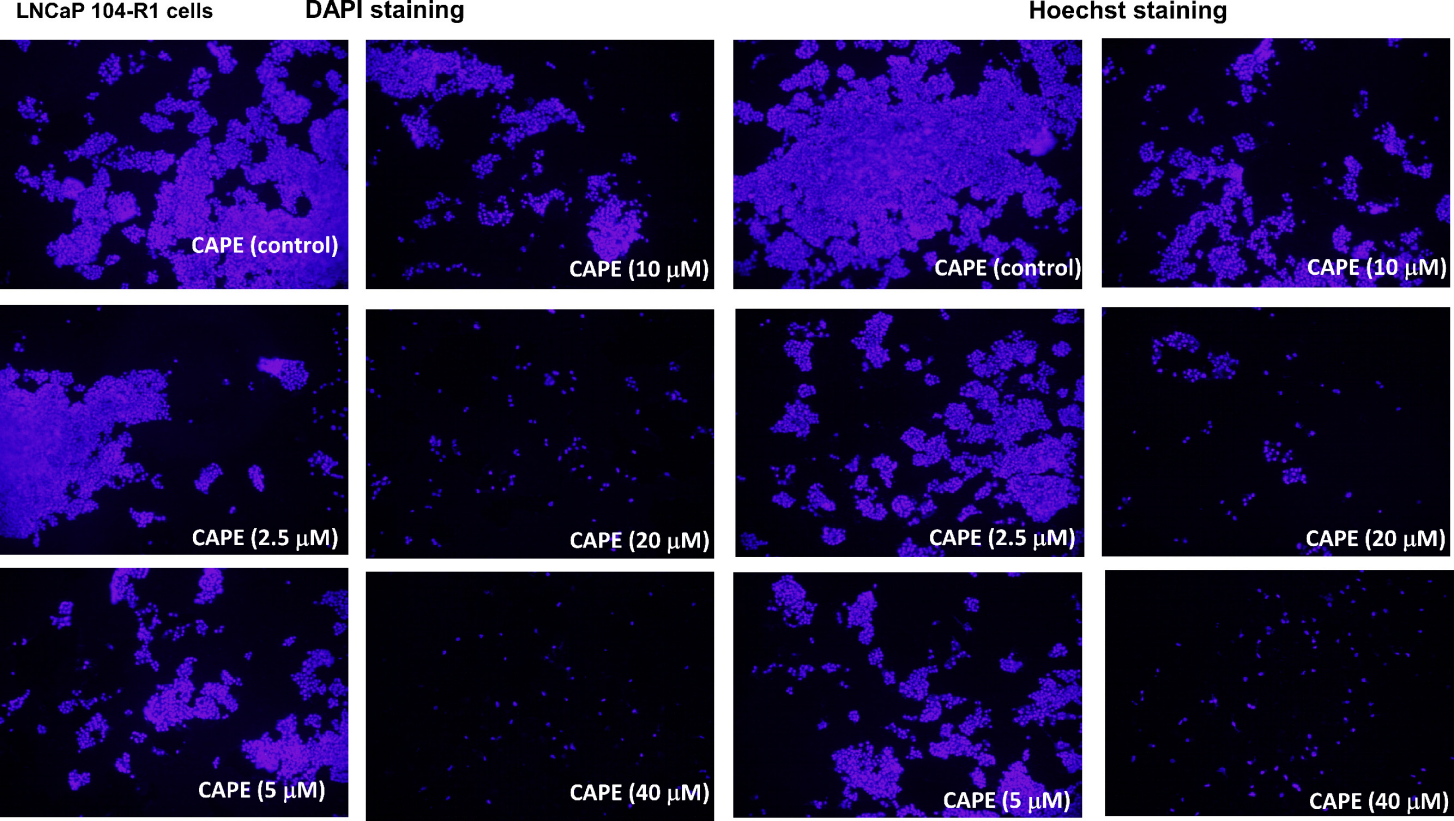
CAPE treatment for 96 h reduced cell proliferation of LNCaP 104-R1 cells DAPI staining and Hoechst dye-staining of LNCaP 104-R1 cells being treated with increasing concentrations of CAPE for 96 h was used to monitor cell proliferation of LNCaP 104-R1 cells using fluorescent microscope with magnification of 100X.

**Figure 2:**
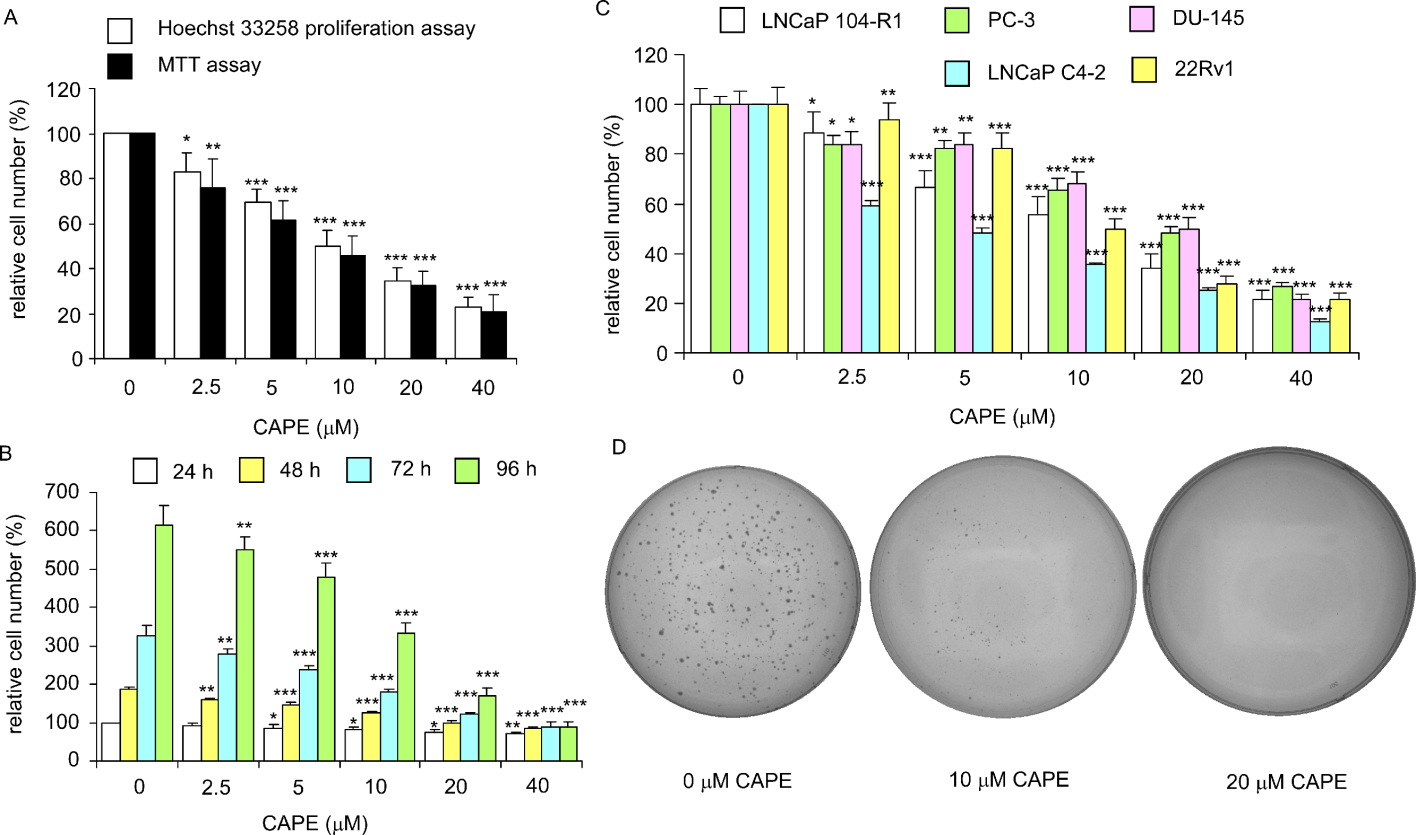
CAPE treatment dose-dependently reduced cell survival, proliferation, and soft agar colony formation of CRPC cells **(A)** LNCaP 104-R1 cells were treated with increasing concentrations of CAPE for 96 h to determine suppressive effect of CAPE on cell proliferation. Relative cell number was determined by either Hoechst 33258 fluorescence based-96 well proliferation assay or by MTT assay. Relative cell number was normalized to cell number of control (no treatment). **(B)** LNCaP 104-R1 cells were treated with increasing concentrations of CAPE for 24, 48, 72, 96 h to investigate the suppressive effects of CAPE. Relative cell number was normalized to cell number of control (no treatment) at 24 h and was determined by Hoechst 33258 fluorescence based-96 well proliferation assay. **(C)** LNCaP 104-R1, PC-3, DU-145, LNCaP C4–2, and 22Rv1 cells were treated with increasing concentrations of CAPE for 96 h to investigate the suppressive effects of CAPE. Relative cell number determined by Hoechst 33258 fluorescence based-96 well proliferation assay and was normalized to cell number of control (no treatment) for individual cell line. Asterisks *, **, and *** represented statistical significance in cell number of *p* < 0.05, *p* < 0.01, and *p* < 0.001, respectively, as compared to that of control. **(D)** Anticancer effect of CAPE was confirmed by the colony formation assay of LNCaP 104-R1 cells treated with 0, 10, or 20 μM CAPE for 14 days. Image is representative of three biological replicates.

Treatment of CAPE (dissolved in ethanol) at 10–40 μM for 96 h significantly reduced the cell number of AR-rich androgen-independent LNCaP 104-R1 cells dose-dependently as determined by light microscopy (Supplementary Figure 1). The ethanol control did not affect cell number of LNCaP 104-R1 cells as compared to no treatment (data not shown). Examination using fluorescent microscopy with Hoechst dye staining and DAPI staining indicated that cell survival and proliferation of commonly used CRPC cell lines, including LNCaP 104-R1 (Figure 1), AR-positive 22Rv1 (Supplementary Figure 2), AR-negative DU-145 (Supplementary Figure 3), and AR-positive LNCaP C4–2 cells (Supplementary Figure 4) were all significantly suppressed by CAPE treatment dose-dependently. The suppressive effects of CAPE on survival of CRPC cells were further confirmed by MTT assay and Hoechst 33258 96-well proliferation assay. MTT assay and Hoechst 33258 proliferation assay indicated an IC_50_ of 16.5 μM and 18.9 μM, respectively, for CAPE to cause growth inhibition on LNCaP 104-R1 cells (Figure 2A). The growth inhibitory effect of CAPE was evident within 24 hours of treatment but the suppressive effect accumulated over time (Figure 2B). The IC_50_ of 24, 48, 72, and 96 h CAPE treatment on LNCaP 104-R1 cells was 64.0, 30.5, 20.5, and 18.0 μM, respectively. We compared the sensitivity of LNCaP 104-R1 cells to CAPE treatment with the four other CRPC cell lines. CAPE treatment dosage-dependently suppressed the proliferation of LNCaP 104-R1, LNCaP C4–2, 22Rv1, PC-3, and DU-145 cells (Figure 2C) with an IC_50_ of 18.9, 10.9, 19.1, 23.2, and 22.6 μM, respectively. CAPE treatment caused the CRPC cells to proliferate slower. The doubling time of LNCaP 104-R1, LNCaP C4–2, 22Rv1, and DU-145 is 30.7, 37.4, 37.4, and 36.0 h, respectively. Under the treatment of 10 μM CAPE, the doubling time of these cells increased to 47.5, 75.8, 106.8, and 40.5 h, respectively. When being treated with 20 μM CAPE, the doubling time of LNCaP 104-R1 and DU-145 further extended to 68.6 and 44.2 h, respectively. We did not examine the doubling time of LNCaP C4–2 and 22Rv1 under treatment of 20 μM CAPE, as they proliferated too slow under this condition. Colony formation assay revealed that treatment with 10 μM CAPE reduced colony formation of LNCaP 104-R1 cells by 90% while treatment with 20 μM CAPE completely blocked the formation of LNCaP 104-R1 colonies (Figure 2D). These results confirmed the anti-cancer effect of CAPE against CRPC cells.

### CAPE treatment induced G1 or G2 cell cycle arrest in CRPC cells

**Figure 3:**
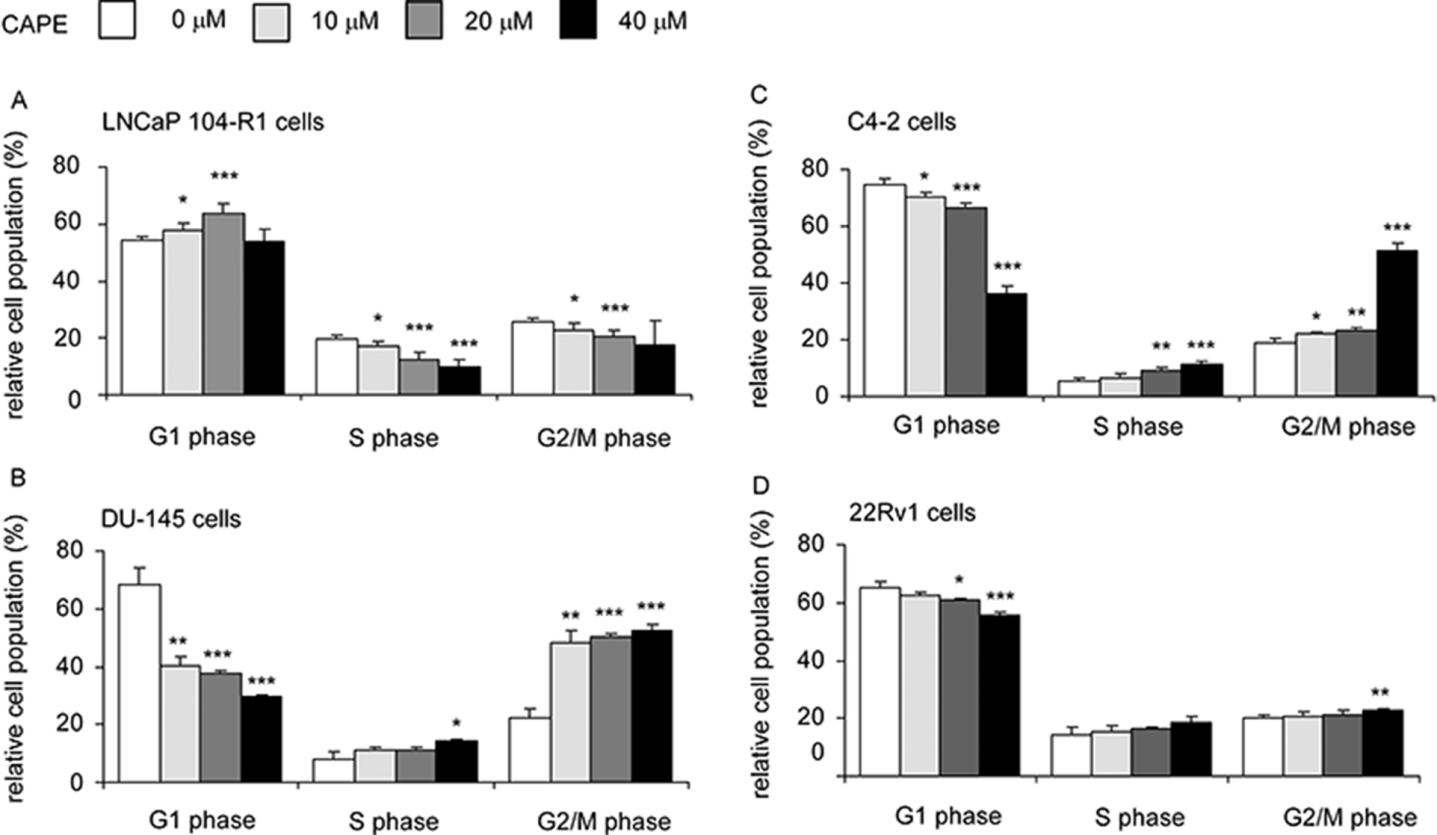
CAPE treatment induced G1 or G2/M cell cycle arrest in CRPC cells LNCaP 104-R1 **(A)**, DU-145 **(B)**, LNCaP C4–2 **(C)**, and 22Rv1 **(D)** cells were treated with 0, 10, 20, or 40 μM CAPE for 96 h, harvested, and stained with propidium iodide dye for flow cytometric analysis of cell cycle distribution. Asterisk* and *** represents statistically significant difference *p* < 0.05 and *p* < 0.001, respectively, between the two group of cells being compared.

Annexin V staining and TUNEL assay for LNCaP 104-R1, LNCaP C4–2, 22Rv1, and DU-145 cells did not reveal any increase of apoptotic cells under CAPE treatment (data not shown). Western blotting analysis illustrated that protein expression of LC3-II and Beclin was not altered by CAPE treatment (data not shown), implying that autophagy probably did not happen in these CRPC cells. Some of the LNCaP 104-R1 cells treated with CAPE showed moderate positive β-galactosidase staining (Supplementary Figure 5). However, the cell morphology did not enlarge, suggesting that CAPE possibly caused hypoxia-induced cell cycle arrest or quiescence in 104-R1 cells, but not cell senescence (Supplementary Figure 5) [23–25]. Flow cytometric analysis revealed a reduction of cells in the S phase and G2/M phase but an increase of cells in the G1 phase population in LNCaP 104-R1 cells under CAPE treatment (Figure 3A), suggesting that CAPE caused G1 cell cycle arrest in LNCaP 104-R1 cells. On the other hand, CAPE treatment reduced G1 phase population but increased G2/M phase population in DU-145 (Figure 3B), LNCaP C4–2 (Figure 3C), and 22Rv1 (Figure 3D) cells, indicating that CAPE caused G2/M cell cycle arrest in DU-145, C4–2, and 22Rv1 cells.

### CAPE treatment retarded the growth of LNCaP 104-R1 xenograft in nude mice

**Figure 4:**
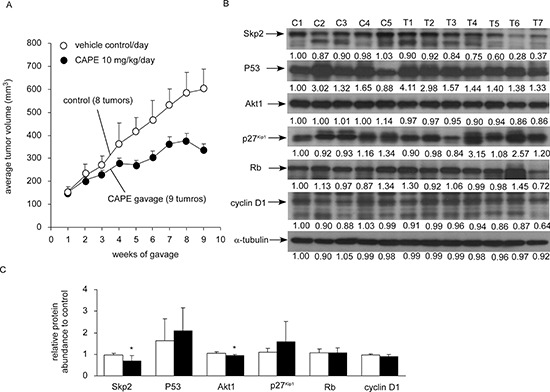
CAPE suppressed tumor growth of LNCaP 104-R1 xenografts **(A)** LNCaP 104-R1 cells were injected subcutaneously into athymic mice to form tumors. After 14 weeks, the average tumor volume exceeded 150 mm^3^. The mice were then separated into control group and CAPE treatment group. Control group contained 6 mice and 8 tumors, while CAPE treatment group contained 6 mice and 9 tumors. CAPE (10 mg/kg/day in sesame oil) or vehicle (sesame oil) was administered by gavage starting from 14th week after cancer cell injection and was shown as 1st week for gavage in figure. Tumor volume and body weight of mice carrying 104-R1 xenografts were measured weekly. Tumor volume was shown as volume plus standard error (SE). Mice body weight in two groups did not show significant difference. **(B)** Protein expression of Skp2, p53, Akt1, p27^Kip1^, cyclin D1, and Rb in LNCaP 104-R1 tumors from control group or CAPE treatment group was assayed with Western blotting assay. α-tubulin was used as loading control. **(C)** The average expression level of Skp2, p53, Akt1, p27^Kip1^, cyclin D1, and Rb proteins in CAPE-treated LNCaP 104-R1 tumors was compared to those in tumors from control group. Asterisk* represents statistically significant difference *p* < 0.05 between the two groups.

Administration of CAPE by gavage (10 mg/kg body weight per day) for eight weeks resulted in 50% reduction of tumor volume (Figure 4A), suggesting that CAPE treatment retarded the growth of LNCaP 104-R1 xenografts. CAPE treatment did not affect the body weight of the mice (data not shown), which means that the dosage used was not overtly toxic. CAPE gavage slowed down the tumor growth of LNCaP 104-R1 cells, which was consistent with our observation that CAPE treatment induced cell cycle arrest but not apoptosis. Western blotting assay indicated that CAPE treatment reduced protein expression of Skp2 and Akt1 in 104-R1 xenografts as compared to the control group (Figure 4B, 4C). Although there was a trend that CAPE increased p53 and p27^Kip1^ but decreased cyclin D1 in tumors, the difference in protein abundance between control and treatment group was not statistically significant (Figure 4C).

### CAPE treatment affected the expression of proteins regulating cell survival, cell proliferation, cell cycle regulation, DNA damage checkpoint, and PI3K-Akt signaling pathway

**Figure 5:**
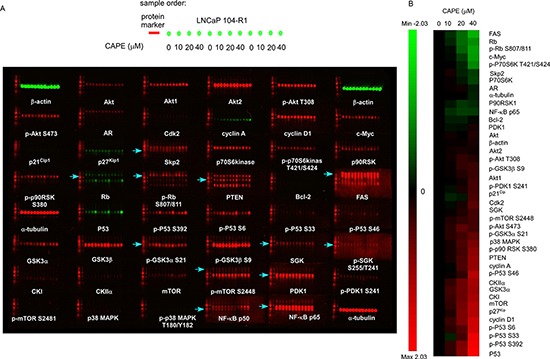
Micro-Western Array image and heatmap of abundance and phosphorylation fold changes of signaling proteins in LNCaP 104-R1 cells treated with CAPE **(A)** LNCaP 104-R1 cells were treated with 0, 10, 20, 40 μM CAPE for 96 h. Micro-Western Arrays were performed to measure the changes in abundance and modification of total Akt, Akt1, Akt2, phospho-Akt Thr308, phospho-Akt Ser473, AR, Cdk2, cyclin A, cyclin D1, c-Myc, p21^Cip1^, p27^Kip1^, Skp2, p70S6 kinase, phospho-p70 S6 kinase Thr421/Ser424, p90RSK, phospho-p90RSK Ser380, Rb, phospho-Rb Ser807/811, PTEN, Bcl-2, fatty acid synthase (FAS), p53, phospho-p53 Ser392, phospho-p53 Ser6, phospho-p53 Ser33, phospho-p53 Ser46, GSK3α, GSK3β, phospho-GSK3α Ser21, phospho-Gsk3β Ser9, SGK, phospho-SGK Ser255/Thr241, CKI, CKIIα, mTOR, phospho-mTOR Ser2448, PDK1, phospho-PDK1 Ser241, phospho-mTOR ser2481, p38 MAPK, phospho-p38 MAPK Thr180/Tyr182, NF-κB p65, and NF-κB p50. Protein abundance of α-tubulin and β-actin was used as loading control. Red color and green color indicated 680 nM and 780 nM wavelength detected by Licor Odyssey scanner for rabbit antibodies and mouse antibodies, respectively. Blue arrows indicated the correct location of band for the detected proteins. **(B)** Proteins were organized in the y-axis of the heatmap based on time of maximal fold change amplitude. Green color indicated decrease of protein expression while red color indicated increase of protein expression under treatment of CAPE.

As CAPE treatment reduced cell proliferation and induced cell cycle arrest in CRPC cells, we used Micro-Western Arrays (MWAs), a high-throughput Western blotting assay [19, 22, 26], to determine how proteins regulating cell proliferation, cell survival, and cell cycle progression are affected by CAPE treatment. CAPE treatment significantly decreased protein levels of fatty acid synthase (FAS), retinoblastoma protein (Rb), phospho-Rb Ser807/811, c-Myc, p70S6kinase, phospho-p70S6kinase Thr421/Ser424, Skp2, p90RSK, and NF-κB p65. Alternatively, CAPE treatment significantly increased p53, phospho-p53 Ser392, phospho-p53 Ser33, phospho-p53 S6, phospho-p53 Ser46, p27^Kip1^, mTOR, CK1, GSK3α, CK2α, cyclin A, p38 MAPK, and p21^Cip1^ (Figure 5A, 5B).

**Figure 6:**
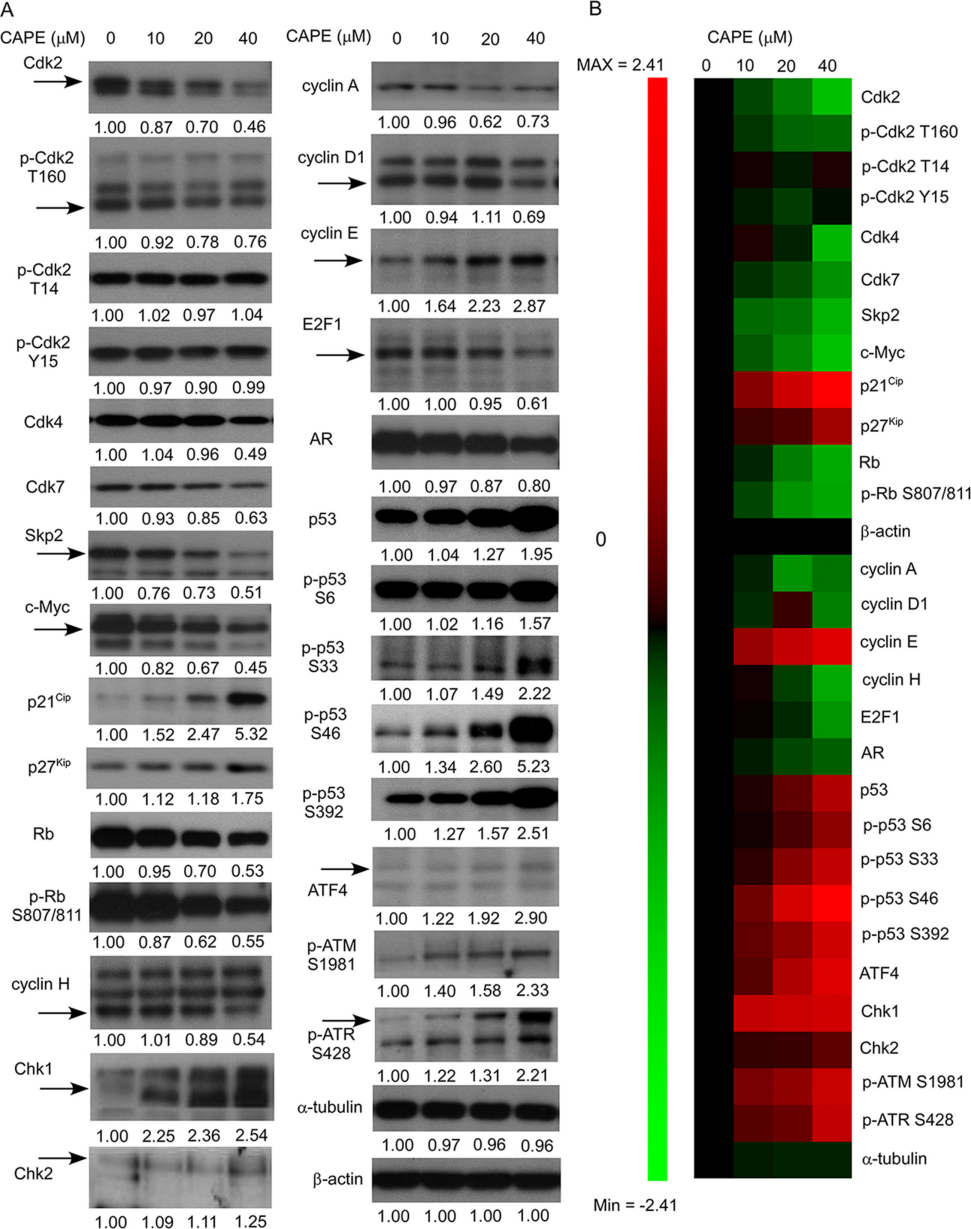
CAPE treatment affected abundance and phosphorylation of proteins regulating proliferation, cell cycle progression, and survival in LNCaP 104-R1 cells **(A)** Protein expression of Cdk2, phospho-Cdk2 Thr160, phospho-Cdk2 Thr14, phospho-Cdk2 Tyr15, Cdk4, Cdk7, Skp2, c-Myc, p21^Cip1^, p27^Kip1^, Rb, phospho-Rb Ser807/811, cyclin H, cyclin A, cyclin D1, cyclin E, E2F-1, AR, p53, phospho-p53 Ser6, phospho-p53 Ser33, and phospho-p53 Ser46, phospho-p53 Ser392, Chk1, Chk2, phospho-ATM S1981, phospho-ATR S428, and ATF4 in LNCaP 104-R1 cells treated with 0, 10, 20, and 40 μM CAPE for 96 h were assayed by Western blotting. Protein abundance of α-tubulin and β-actin was used as loading control. **(B)** Proteins expression level was organized in the y-axis of the heatmap based on time of maximal fold change amplitude. Green color indicated decrease of protein expression while red color indicated increase of protein expression under treatment of CAPE.

Conventional Western blotting assay was then used to confirm the changes of protein expression. CAPE treatment affected proteins regulating cell cycle, proliferation, survival, DNA damage check point, and PI3K-Akt signaling pathway. Expression of Cdk2, phospho-Cdk2 Thr160, Cdk4, Cdk7, Skp2, c-Myc, Rb, phospho-Rb Ser807/811, cyclin A, cyclin D1, cyclin H, and E2F1 proteins was significantly suppressed by CAPE treatment (Figure 6A, 6B), while protein abundance of cyclin E, p27^Kip1^, p21^Cip1^, p53, phospho-p53 Ser392, phospho-p53 Ser33, phospho-p53 S6, phospho-p53 Ser46, CHK1, CHK2, phospho-ATM Ser1981, phospho-ATR Ser428, and ATF4 (Figure 6A, 6B) were significantly induced by CAPE treatment.

**Figure 7:**
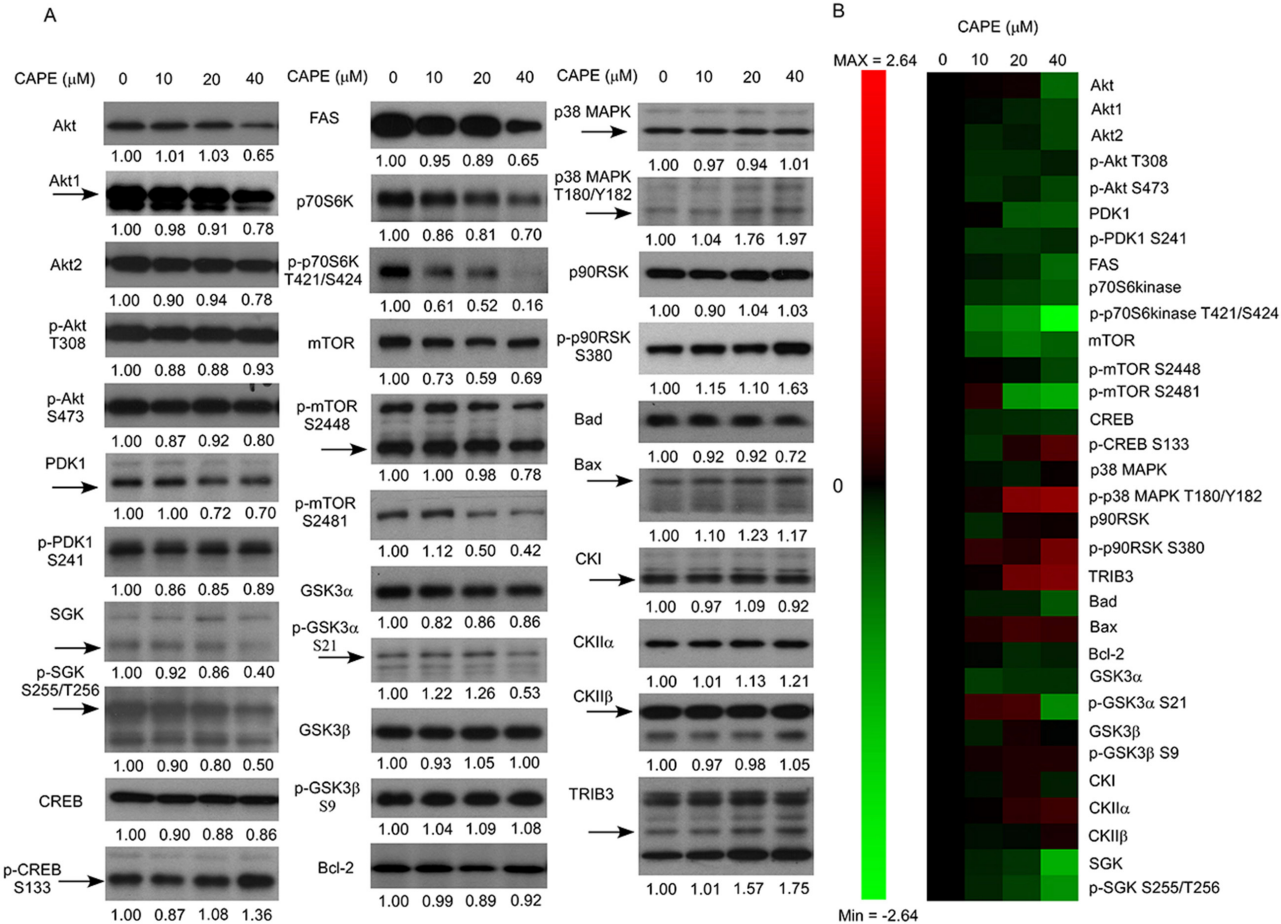
CAPE treatment affected abundance and phosphorylation of proteins involved in PI3K-Akt signaling pathways **(A)** Protein expression of Akt, Akt1, Akt2, phospho-Akt Thr308, phospho-Akt Ser473, PDK1, phospho-PDK1 Ser241, SGK, phospho-SGK Ser255/Thr256, CREB, phospho-CREB Ser133, FAS, p70S6 kinase, phospho-p70 S6 kinase Thr421/Ser424, mTOR, phospho-mTOR Ser2448, phospho-mTOR Ser2481, GSK3α, phospho-GSK3α Ser21, GSK3β, phospho-GSK3β Ser9, Bcl-2, p38 MAPK, phospho-p38 MAPK Thr180/Tyr182, p90RSK, phospho-p90RSK S380, Bad, Bax, CKI, CKIIα, CKIIβ, Chk1, Chk2, phospho-ATM S1981, phospho-ATR S428, and TRIB3 in LNCaP 104-R1 cells treated with 0, 10, 20, and 40 μM CAPE for 96 h were assayed by Western blotting. Protein abundance of α-tubulin and β-actin was used as loading control. **(B)** Proteins expression level was organized in the y-axis of the heatmap based on time of maximal fold change amplitude. Green color indicated decrease of protein expression while red color indicated increase of protein expression under treatment of CAPE.

The protein abundance of total Akt, Akt1, Akt2, and phospho-Akt Ser473 was decreased by CAPE treatment (Figure 7A, 7B). Additionally, CAPE treatment suppressed the protein expression of PDK1, SGK, phospho-SGK S255/T256, FAS, p70S6kinase, phospho-p70S6kinase Thr421/Ser424, mTOR, phospho-mTOR Ser2481, and phospho-GSK3α Ser21 (Figure 7A, 7B). Conversely, CAPE treatment increased phospho-CREB Ser133, phospho-p38 MAPK Thr180/Tyr182, and phospho-p90RSK Ser380, Bax, CKIIα, and TRIB3.

**Figure 8:**
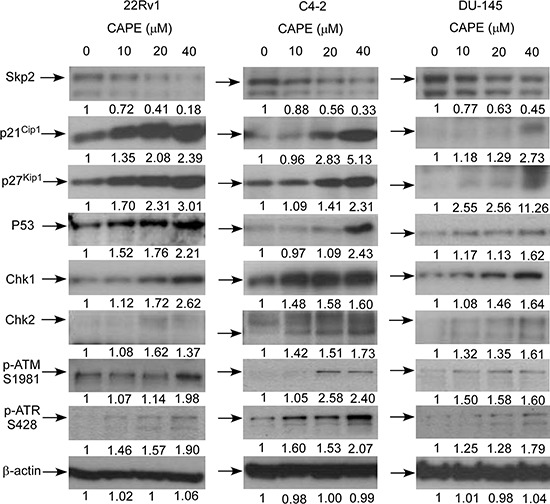
CAPE treatment affected abundance of proteins involved in cell cycle regulation and DNA damage checkpoint in CRPC cells Protein expression of Skp2, p21^Cip1^, p27^Kip1^, p53, Chk1, Chk2, phospho-ATM S1981, phospho-ATR S428 in 22Rv1, LNCaP C4–2, and DU-145 cells treated with 0, 10, 20, and 40 μM CAPE for 96 h were assayed by Western blotting. Protein abundance of β-actin was used as loading control.

**Figure 9:**
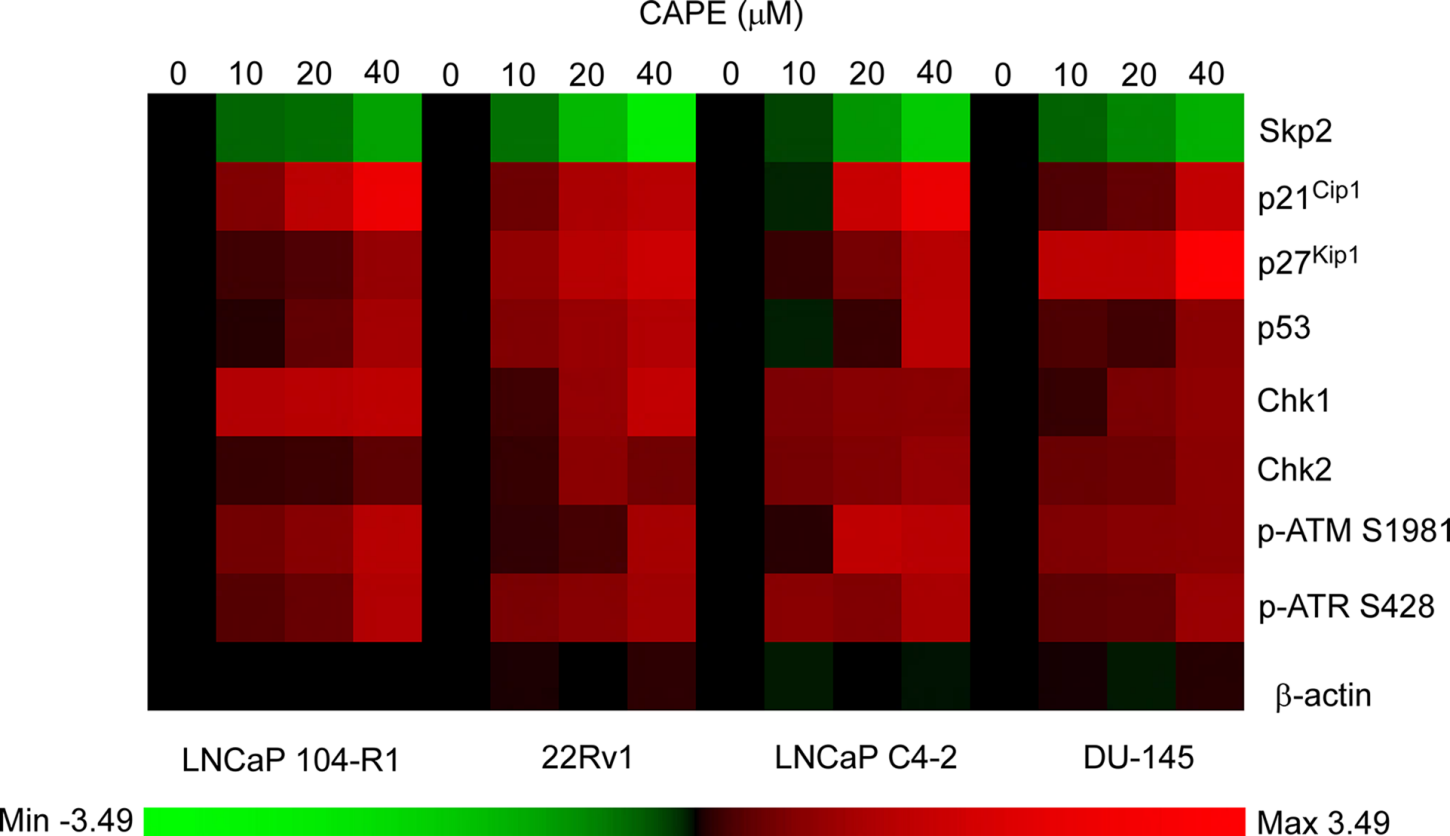
Expression pattern of cell cycle regulation and DNA damage checkpoint proteins in CRPC cells being treated with CAPE Proteins expression of Skp2, p21^Cip1^, p27^Kip1^, p53, Chk1, Chk2, phospho-ATM S1981, phospho-ATR S428 in LNCaP 104-R1, 22Rv1, LNCaP C4–2, and DU-145 cells treated with 0, 10, 20, and 40 μM CAPE for 96 h assayed in Figure 6 and Figure 8 was organized in the y-axis of the heatmap based on time of maximal fold change amplitude. Green color indicated decrease of protein expression while red color indicated increase of protein expression under treatment of CAPE.

Skp2, p21^Cip1^, p27^Kip1^, and p53 are proteins important in regulating cell proliferation and cell cycle progression, while Chk1, Chk2, ATM, and ATR are DNA damage checkpoint proteins. Expression of these proteins was significantly affected by CAPE treatment in LNCaP 104-R1 cells (Figures 5–7). We therefore examined if CAPE treatment also affected expression of these proteins in 22Rv1, LNCaP C4–2, and DU-145 cells. Similar to LNCaP 104-R1 cells, Skp2 protein abundance in 22Rv1, C4–2, and DU-145 cells was significantly suppressed by CAPE treatment (Figure 8, Figure 9). On the other hand, CAPE treatment induced expression of p21^Cip1^, p27^Kip1^, p53, Chk1, Chk2, phospho-ATM Ser1981, and phospho-ATR Ser428 in all four CRPC cell lines (Figures 8, 9). The changes of these proteins may all contribute to the inhibition of cell growth as well as induction of cell cycle arrest in CRPC cells.

### Overexpression of Skp2 rescued the suppressive effect of CAPE on cell proliferation of 104-R1 cells

**Figure 10:**
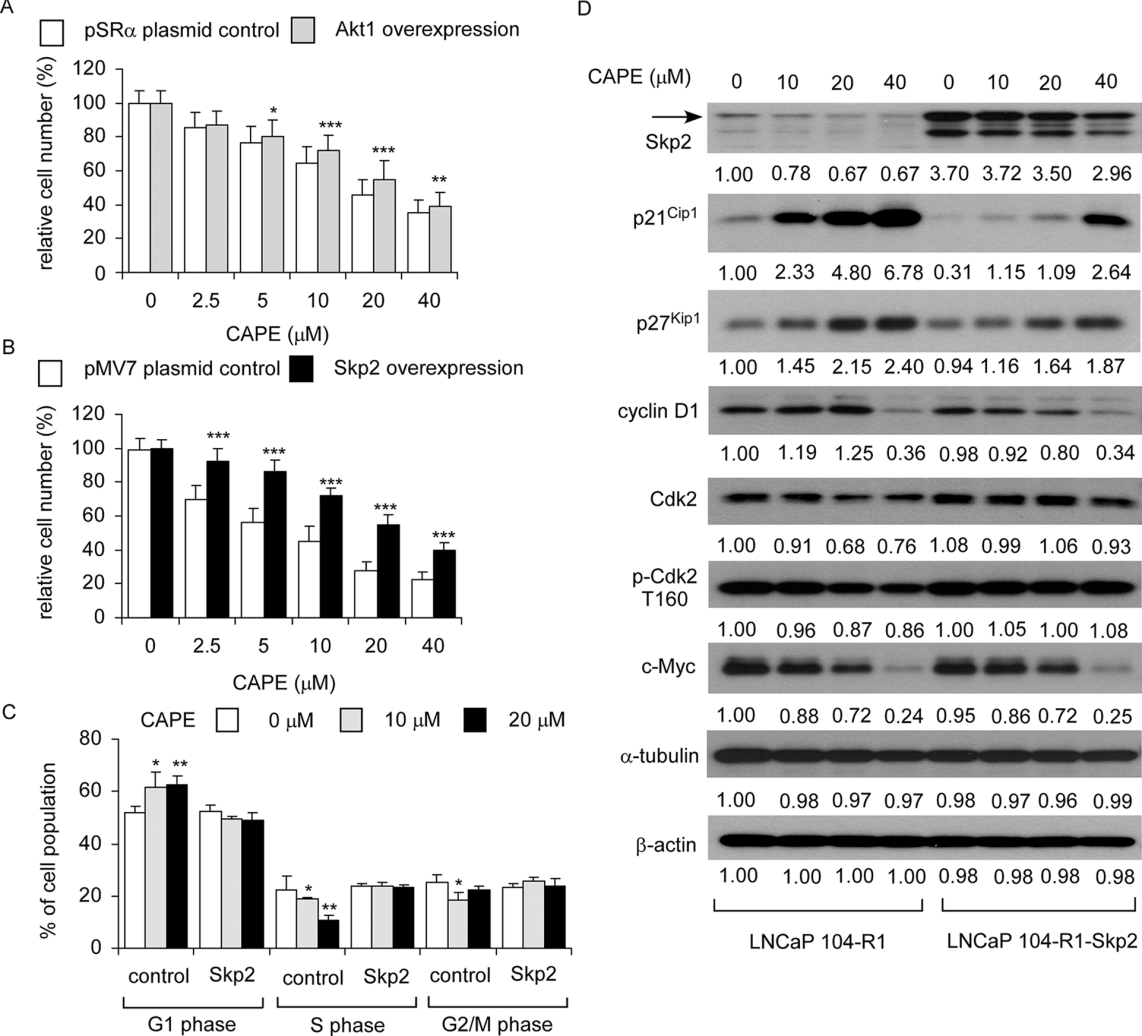
Over-expression of Skp2 blocked the suppressive effect of CAPE on proliferation of LNCaP 104-R1 cells LNCaP 104-R1 cells overexpressing Akt1 **(A)** or Skp2 **(B)** and their plasmid control cells were treated with increasing concentrations of CAPE for 96 hr and analyzed by 96-well proliferation assay for cell proliferation. **(C)** LNCaP 104-R1 cells overexpressing with Skp2 or control plasmid were treated with 0, 10, or 20 μM CAPE for 96 h, harvested, and stained with propidium iodide dye for flow cytometric analysis of cell cycle distribution. **(D)** Protein expression of Skp2, p21^Cip1^, p27^Kip1^, cyclin D1, Cdk2, phospho-Cdk2 T160, and c-Myc were assayed by Western blotting in LNCaP 104-S cell lines overexpressing Skp2 or vector control prior to CAPE treatment. Protein abundance of α-tubulin and β-actin was used as loading control.

CAPE treatment caused 49%, 55%, and 65% reduction of Skp2, c-Myc, and total Akt, respectively. To determine if CAPE suppressed cell proliferation through suppression of Skp2, c-Myc, or Akt, we overexpressed Skp2, c-Myc, and Akt1 in LNCaP 104-R1 cells. Akt1 over-expression slightly blocked the anti-proliferative effect of CAPE (Figure 10A). Surprisingly, c-Myc over-expression did not show any rescue effect (data not shown). Overexpression of Skp2 in LNCaP 104-R1 cells significantly blocked the suppressive effect of CAPE treatment (Figure 10B). Flow cytometry analysis indicated that CAPE treatment induced G1 cell cycle arrest in control LNCaP 104-R1 cells but not LNCaP 104-R1 cells overexpressing Skp2 (Figure 10C). CAPE treatment suppressed cyclin D1 and c-Myc in both control and Skp2 overexpressing LNCaP 104-R1 cells (Figure 10D). However, the accumulation of p27^Kip1^ and p21^Cip1^ was 22% and 50% less, respectively, while abundance of Cdk2 and phospho-Cdk2 Thr160 were either less or not affected by CAPE in Skp2 overexpressing LNCaP 104-R1 cells as compared to the control LNCaP 104-R1 cells. The lower endogenous level p27^Kip1^ in Skp2 overexpressing LNCaP 104-R1 cells was consistent to the function of Skp2 as Skp2 target p27^Kip1^ for degradation. Consequently, reduction of Skp2, Cdk2, and phospho-Cdk2 Thr160, as well as accumulation of p27^Kip1^ and p21^Cip1^ were likely to play essential roles in the G1 cell cycle arrest induced by CAPE treatment in LNCaP 104-R1 cells.

### Knockdown of p27^Kip1^, p21^Cip1^, or p53 rescued the suppressive effect of CAPE on cell proliferation of LNCaP 104-R1 cells

**Figure 11:**
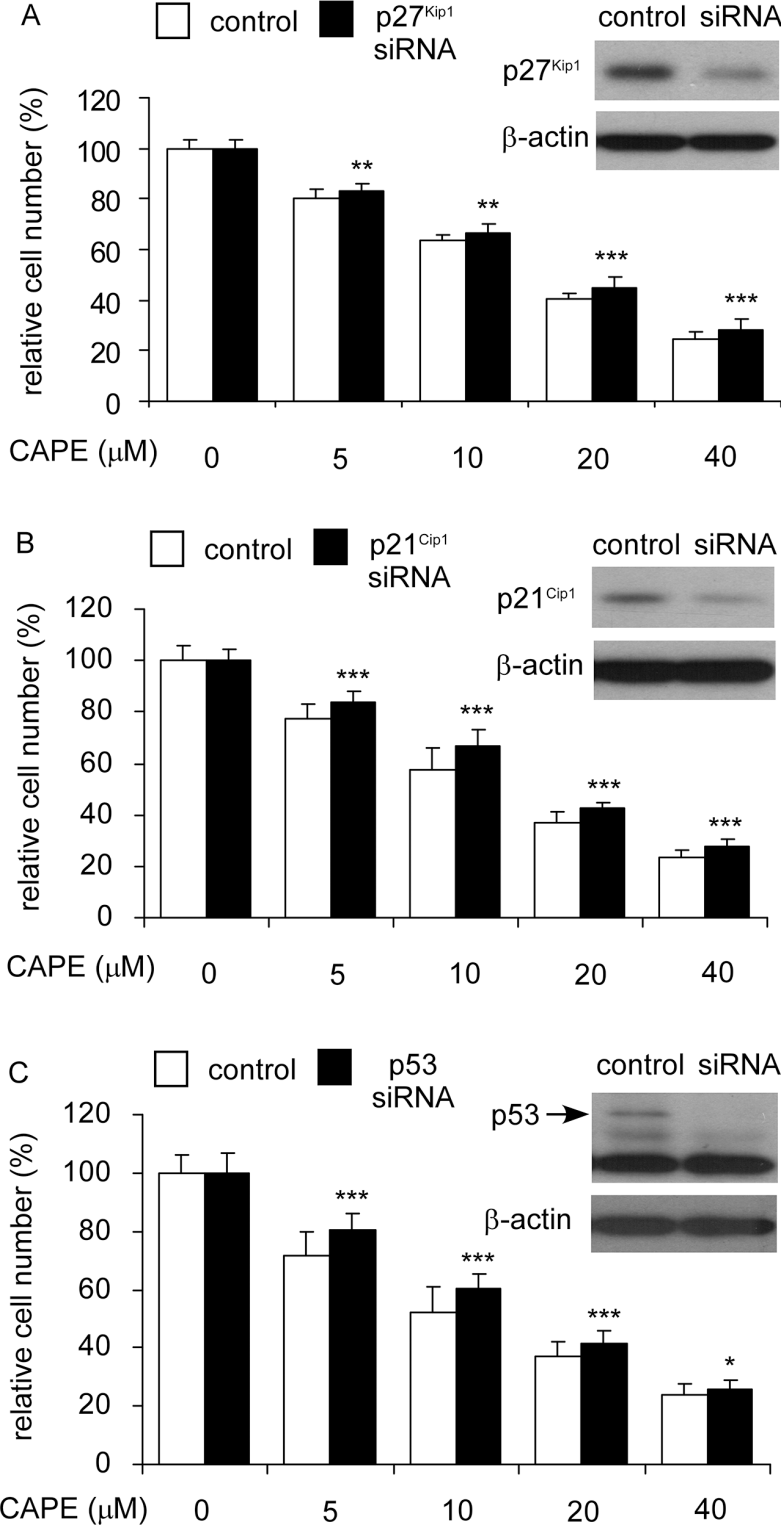
Over-expression of p27Kip1, p21Cip1, and p53 blocked the suppressive effect of CAPE on proliferation of LNCaP 104-R1 cells LNCaP 104-R1 cells overexpressing p27^Kip1^ **(A)**, p21^Cip1^ **(B)**, or p53 **(C)** and their plasmid control cells were treated with increasing concentrations of CAPE for 96 hr and analyzed by 96-well proliferation assay for cell proliferation. Overexpression of p27^Kip1^, p21^Cip1^, and p53 proteins was confirmed by Western blotting. Protein abundance of β-actin was used as loading control.

Besides Skp2, CAPE treatment induced protein expression of p27^Kip1^, p21^Cip1^, and p53 in all CRPC cell lines. We therefore determined if siRNA knockdown of p27^Kip1^, p21^Cip1^, or p53 may rescue the growth inhibition of LNCaP 104-R1 cells induced by CAPE treatment. Indeed, siRNA knockdown of p27^Kip1^ (Figure 11A), p21^Cip1^ (Figure 11B), or p53 (Figure 11C) rescued the cell proliferation of LNCaP 104-R1 cells being treated with increasing concentration of CAPE, confirming their essential role in regulating cell cycle arrest induced by CAPE treatment.

### Co-treatment of CAPE with LY294002 or ABT737 suppressed proliferation of LNCaP 104-R1 cells

**Figure 12:**
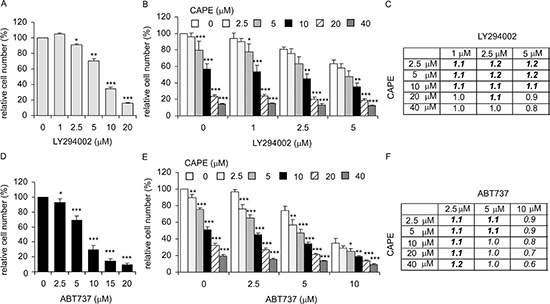
Combined treatment of CAPE with PI3K inhibitor LY294002 or BCl-2 inhibitor ABT737 showed additive and mild synergistic inhibition on proliferation of LNCaP 104-R1 cells Proliferation of LNCaP 104-R1 cells treated with increasing dosage (0, 5, 10, 20 μM) of LY294002 **(A)**, combination of CAPE and LY294002 **(B)**, ABT737 **(D)**, and combination of CAPE with ABT737 **(E)** was determined by 96-well proliferation assay. The ration of expected cell number/observed cell number of LNCaP 104-R1 cells treated with combination of CAPE and LY294002 **(C)** or combination of CAPE and ABT737 **(F)** was shown. The effect of the combined treatment was determined by the ratio of expected cell number/observed cell number. For example, treatment of 104-R1 cells with 5 μM of ABT737 decreased the cell number to 69.2% and treatment with 104-R1 cells with 5 μM CAPE alone decreased the cell number to 75.6%. The expected cell number of treatment combining 5 μM of ABT737 with 5 μM CAPE was 0.692 × 0.756 = 52.3%. The actual observed cell number is 47.0%. The ratio of expected cell number/observed cell number is 0.523/0.470 = 1.1. Ratio larger than one represents synergy of growth inhibition as the combined treatment of two drugs suppressed more cells than either drug alone. If the observed cell number is less than the cell number being treated with any one of the drug alone, this indicates additive suppressive effect of the combination treatment of the two drugs. For example, treatment of 104-R1 cells with 2.5 μM of LY294002 decreased the cell number to 91.2%, while treatment with 104-R1 cells with 40 μM CAPE decreased the cell number to 14.5%. The combination of 2.5 μM of LY294002 with 40 μM CAPE decreased the cell number to 13.1%, then we called the combination of 2.5 μM of LY294002 with 40 μM CAPE exhibited additive suppressive effects on LNCaP 104-R1 cells.

Synergistic effect implies the suppressive effect of two drugs being treated together is greater than the sum of their separate suppressive effect at the same doses. Additive suppressive effect indicates that the combination of two drugs produces an effect that is greater than the effect of one of the drug. According to the fact that phosphorylation of Akt was only slightly suppressed by CAPE treatment and overexpression Akt1 only slightly rescued the suppressive effect of CAPE treatment in LNCaP 104-R1 cells, we hypothesized that co-treatment of CAPE with PI3K inhibitor LY294002 will show additive suppressive effect on cell growth of LNCaP 104-R1 cells. Indeed, combination of 2.5–10 μM CAPE with 1–5 μM LY294002 showed synergetic suppression on cell growth of LNCaP 104-R1 cells (Figure 12A–12C). Combination of higher dose of CAPE (20 or 40 μM) with LY294002 only showed additive but not synergistic suppressive effects. Expression of Bcl-2 in androgen-independent LNCaP 104-R1 cells is 65-fold higher that in parental androgen-dependent LNCaP 104-S cells at culture condition [21]. Bcl-2 expression may protect LNCaP 104-R1 cells from cell death under stress or drug treatment. CAPE treatment slightly decreased the protein level of Bcl-2 (Figure 7). We anticipated that co-treatment with Bcl-2 inhibitor ABT737 will exhibit additive suppressive effect. As shown in Figure 12D–12F, combined treatment of CAPE with ABT737 demonstrated additive suppressive effect while combination of low dose of ABT737 with CAPE displayed synergetic suppressive effect.

### Clinical implication of p53 induction

**Figure 13:**
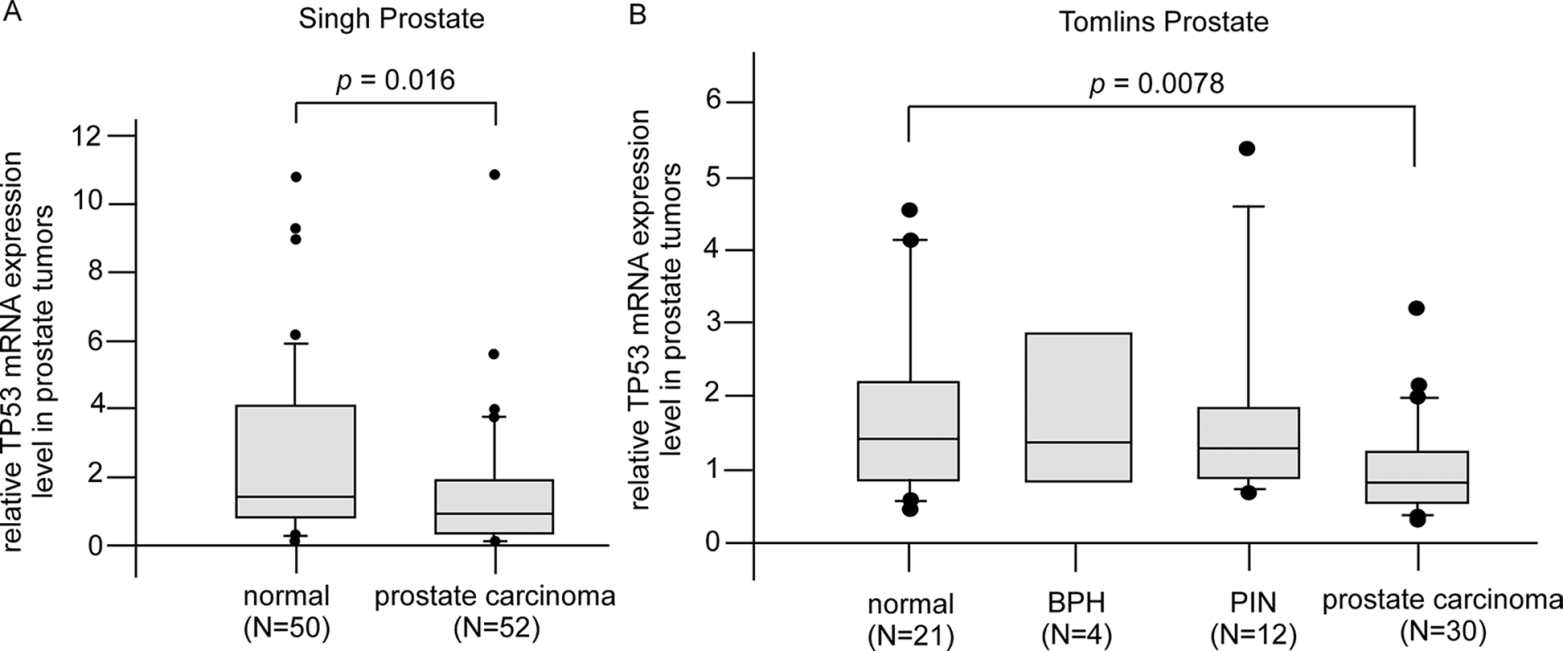
Gene expression of Tp53 in PCa patient oncomine database **(A)** Expression of Tp53 gene was detected by reporter probe 1974_s_at in 50 normal prostate gland samples and 52 prostate carcinoma samples from Singh prostate datasets using gene microarray [108]. **(B)** Expression of Tp53 gene was detected by reporter probe IMAGE:24415 in 21 normal prostate gland epithelial samples, 4 BPH (benign prostatic hyperplasia), 12 PIN (prostatic intraepithelial neoplasia), and 30 prostate carcinoma samples from Tomlins prostate datasets using gene microarray [109]. Data were downloaded from Oncomine (http://www.oncomine.com) without further processing.

Analysis of Oncomine database suggested that PCa tumors expressed less Tp53 as compared to normal prostate epithelial tissues (Figure 13A, 13B). Since CAPE treatment significantly increased abundance of p53 protein, CAPE treatment is thus a potential effective therapy for PCa.

## DISCUSSION

Our observations implied that CAPE treatment at dosage 10–20 μM can effectively suppressed the proliferation, survival, soft agar colony formation, and tumor growth of CRPC cells via induction of G1 or G2/M cell cycle arrest. The achievable physiological concentration of CAPE in human serum is 17 μM [27], therefore, administration of CAPE is a possible treatment for CRPC. CAPE is distributed extensively into animal tissues and is eliminated rapidly with a short half life [28]. Toxicology study revealed that i.p. injection of 10 mg/kg of CAPE did not show any toxicity on liver or kidney in mice study while i.p. injection of higher dose (20 and 30 mg/kg) CAPE caused mild dose-dependent toxicity on liver and kidney [29]. CAPE treatment has also been shown to sensitize cancer cells to chemotherapeutic drugs and radiation treatment [30]. Therefore, treatment with CAPE not only may suppress CRPC tumor growth in patients but may also protect PCa patients from chemotherapy or radiation therapy.

The p53 protein is encoded by the Tp53 gene. The p53 protein is a main regulator of the cell cycle arrest and cellular senescence in response to short telomeres, DNA damage, oncogenes, supraphysiological mitogenic signals, and tumor suppressor gene overexpression [31]. The p53 protein is negatively regulated by the E3 ubiquitin-protein ligase HDM2, which facilitates its degradation [32]. HDM2 is negatively regulated by the alternate-reading-frame protein (ARF) [32]. The p53 protein establishes the cell cycle arrest in part by inducing the expression of p21^Cip1^ [32]. The ATR kinase is a key transducer of genomic damage induced by oncogenes [33]. Activation of ATR is sufficient to promote cell cycle arrest and, if persistent, triggers p53-dependent but p16/ARF-independent senescence [34]. CAPE treatment can alter redox state and induce DNA damage in cancer cells [35]. ATR is a serine/threonine-specific protein kinase which is the sensor for DNA damage [36–38]. ATR activates the DNA damage checkpoint, which leads to cell cycle arrest [33, 37, 38]. Persistent single-stranded DNA activates ATR [33, 37, 38]. Activated ATR then phosphorylates Chk1, initiating a signal transduction cascade that culminates in cell cycle arrest [33]. ATM (Ataxia telangiectasia mutated) is a serine/threonine protein kinase which is recruited and activated by DNA double-strand breaks. It phosphorylates p53, Chk2, H2AX, and other tumor suppressors, which initiates the activation of the DNA damage checkpoint and leads to cell cycle arrest, DNA repair or apoptosis, [39]. Upon DNA damage, ATM autophosphorylates on residue Ser1981, stimulating the dissociation of ATM dimmers and is therefore followed by the release of active ATM monomers [40]. The ATM-mediated DNA damage response consists of both the rapid and the delayed response. ATM phosphorylates and activates the effector kinase Chk2 [41, 42]. Activated Chk2 then phosphorylates phosphatase CDC25A, which is degraded and is unable to dephosphorylate Cdk2-Cyclin, resulting in cell-cycle arrest [41, 42]. If the DSB can not be repaired during this rapid response, ATM with then phosphorylate MDM2 and p53 at Ser15 [41, 42]. p53 is also phosphorylated by the effector kinase Chk2 [41, 42]. These phosphorylation finally lead to stabilization and activation of p53 and subsequent transcription of several p53 target genes including p21^Cip1^ and therefore induce long-term cell-cycle arrest or apoptosis [41, 42]. Chk1 is a serine/threonine protein kinase and is a key regulator of genome stability, cell cycle, and cell survival [43]. Chk1 coordinates the DNA damage response [44]. Chk1 is regulated by ATR through phosphorylation. Activation of Chk1 results in the initiation of cell cycle checkpoints, cell cycle arrest, DNA repair, or even apoptosis [44–46]. Activation of Chk1 holds the cell in the G2 phase until ready to enter the mitotic phase. Chk1 is also essential for the cell to enter S or M phase [45, 46]. Chk2, is a protein kinase that is activated in response to DNA damage and is involved in cell cycle arrest [47]. Activated Chk2 inhibits Cdc5c phosphatase, prevents entry into mitosis phase, stabilizes the tumor suppressor protein p53, and leads to G1 cell cycle arrest [48]. CAPE treatment significantly reduced protein abundance of Skp2 and induced protein level and phosphorylation of p53, ATM, and ATR, as well as the abundance of p21^Cip1^, p27^Kip1^, Chk1, and Chk2 protein in CRPC cells (Figures 6, 8, 9). These changes may contribute to the induction of cell cycle arrest in CRPC cells.

As mentioned by Dr. Blagosklonny, cell cycle arrest is not yet senescence [23, 24]. When the cell cycle is arrested, an inappropriate growth-stimulation, such as activation of mTOR, converts the cell cycle arrest into cellular senescence [23, 24]. Properties of cellular senescence include a large flat morphology, SA-β-gal staining, feedback signal resistance, and loss of regenerative potential [24]. According to the facts that CRPC cells treated with CAPE did not show enlargement, and CAPE treatment suppressed mTOR signaling related proteins (Figure 7), only a portion of cells showed positive SA-β-gal staining, and some cells still proliferate under CAPE treatment, we believe that CAPE treatment induced quiescence cell cycle arrest but not cellular senescence. Hypoxia can increase content and functions of lysosomal, which may display as moderate SA-β-Gal-staining in cells [25]. In some cells, hypoxia can slow down cell proliferation and cause cell cycle arrest [25]. Under hypoxic conditions, cells are relatively small, whereas senescent cells are large and flat [25]. Hypoxia-arrested cells can resume proliferation when being placed under normoxia [25]. We therefore believe that CAPE treatment triggered hypoxia-induced cell cycle arrest/quiescence in LNCaP 104-R1 cells.

Skp2 is an F-box protein belongs to the SCF (Skp1-Cullin 1-F-box protein) E3 ubiquitin ligase complex which regulates the S phase entry of cells by inducing the degradation of the cyclin-dependent kinase (Cdk) inhibitors p21^Cip1^, p27^Kip1^, p57, p130, Tob1, and FoxO1 [49–51]. Skp2 targets Cdk inhibitor p27^Kip1^ by phosphorylating p27^Kip1^ at T187 for ubiquitination and degradation [52–54]. Both luminal and basal epithelial cells in normal prostate exhibit very low Skp2 levels, however, Skp2 levels increase dramatically in both prostatic intraepithelial neoplasm (PIN) and PCa [49, 55]. Up-regulation of Skp2 correlates to lower p27^Kip^ expression, higher Gleason score, more advanced pathological stage, and recurrence in PCa patients [55–57]. Up-regulation of Skp2 in PCa patient is an independent factor for prediction of higher risk of PCa recurrence after surgery [55, 56]. Skp2 overexpression in PCa cells stimulates PCa cell proliferation and increases the tumorigenesis in xenograft tumor model [58]. Tissue-specific over-expression of Skp2 in prostate promotes proliferation, hyperplasia, dysplasia, and low-grade carcinoma in the prostate gland [59]. Deficiency of Skp2 *in vivo* triggers cellular senescence via up-regulation of p21^Cip1^, p27^Kip1^, and ATF4, therefore suppresses the development of PCa [60]. Skp2 was reported to cross-talk with PI3K/Akt [61], AR [62], PTEN [55], and BRCA2 [63] signaling pathways in PCa cells. As a result, Skp2 plays essential role in the development and progression of human PCa [49]. Development of compounds targeting Skp2 may be a useful strategy for the treatment of patients with CRPC. We discovered that overexpression of Skp2 reduced the accumulation of p21^Cip1^and p27^Kip1^ as well as lessen the decrease of Cdk2 and phospho-Cdk2 T160 caused by CAPE treatment (Figure 10). CAPE treatment reduced protein expression of Skp2 but induced protein abundance of p21^Cip1^, p27^Kip1^, p53, and ATF4 (Figures 4–6, 8, 9). Changes of these proteins may contribute to the induction of cell cycle arrest in CRPC cells.

Cyclin A is a member of the cyclin family. Transcription of cyclin A is tightly regulated and synchronized with cell cycle progression by the transcription factor E2F in a negative feedback loop [64]. Both cyclin A and E2F were suppressed by CAPE treatment (Figure 6). Cdk2 is a member of the cyclin-dependent kinase family of serine/threonine protein kinases [65]. Complex of Cdk2 and cyclin A is required to progress through the S phase, while binding between Cdk2-cyclin E is required for the transition of cells from G1 to S phase [65]. Activation of Cdk2 complexes requires phosphorylation of Thr160 on Cdk2 by Cdk7 and cyclin H [66] as well as dephosphorylation of Thr14 and Tyr15 on Cdk2 by cdc25 phosphatase. Although CAPE treatment did not alter phosphorylation of Thr14 and Tyr 15 on Cdk2, it repressed phosphorylation of Thr160 on Cdk2 (Figure 6), which will suppress the activity of Cdk2. Skp2 is phosphorylated by Cdk2 at Ser64 [54] and by Akt at Ser72 [67]. Phosphorylation of Ser64 and Ser72 on Skp2 regulates the stabilization of Skp2 by preventing its association with APC/CCdh1 [51, 52, 54, 67]. Protein abundance and phosphorylation of Cdk2 and Akt were both declined by CAPE treatment (Figures 6, 7). CAPE treatment may therefore reduce the stability of Skp2, resulting in reduction of Skp2 protein abundance. Cdk4 is a serine/threonine protein kinase which is important for cell cycle G1 phase progression [68]. The activity of Cdk4 is controlled by CDK inhibitor p16^INK4a^. Cdk4 is responsible for the phosphorylation of retinoblastoma (Rb) [68]. CAPE treatment suppressed abundance of Cdk4, Rb, and phosphor-Rb Ser807/811 (Figure 6). Complex between Cyclin D and Cdk4 or Cdk6 are key player for G1/S transition in cell cycle progression [69]. Expression of cyclin D1 was significantly reduced by CAPE treatment. As a result, CAPE treatment may interfere the cell cycle progression and induced G1 or G2/M cell cycle arrest by suppressing the protein abundance and activity of Skp2, Cdk2, Cdk4, Cdk7, cyclin A, cyclin D1, cyclin H, E2F1, and c-Myc as well as by inducing p21^Cip1^ and p27^Kip1^.

Phosphatase and tensin homolog (PTEN) protein is a negative regulator for PI3K-Akt signaling pathway [70]. PTEN is frequently deleted or mutated in prostatic intraepithelial neoplasia (PIN) and PCa, giving rise to elevation of phosphoinositide 3-kinase (PI3K)/Akt signaling [71, 72]. Up-regulation of PI3K/Akt activity is associated with poor clinical outcome of PCa [72–78]. Akt is a serine/threonine protein kinase with three isoforms, the Akt1, Akt2, and Akt3 [79, 80]. Two phosphorylation sites on Akt, threonine 308 and serine 473, regulate activity of Akt. Phosphorylation of Thr308 on Akt is activated by PDK1 [81], while the phosphorylation of serine 473 on Akt is activated by mTOR kinase, its associated protein rector, and SIN1/MIP1 [82, 83]. Akt phosphorylation level correlates with higher Gleason score [84, 85]. CAPE treatment caused mild but dose-dependent repression of Akt1, Akt2, as well as phosphorylation of Akt and PDK1 (Figure 7). This explained why co-treatment of (PI3K)-Akt inhibitor LY294002 showed synergistic suppressive effect (Figure 12). The mammalian target of rapamycin (mTOR) is also a serine/threonine protein kinase [86, 87]. The mTOR protein is phosphorylated at Ser2448 via the PI3 kinase/Akt signaling pathway and autophosphorylated at Ser2481 [88, 89]. CAPE treatment significantly suppressed the phospho-mTOR Ser2481 and phospho-p70S6kinase, a downstream signaling protein of mTOR pathway [90, 91], while caused a relatively mild suppression on mTOR and phosphor-mTOR Ser2448 (Figure 7). As mTOR and p70 S6 kinase function as ATP and amino acid homeostasis sensor, regulator protein synthesis, balance nutrient uptake, and control cell proliferation [92, 93], their inhibition caused by CAPE treatment may interfere nutrient balance, protein synthesis, and diminish cell proliferation in CRPC cells. TRIB3 is a putative protein kinase and is induced by NF-κB [94, 95]. TRIB3 is a negative regulator of NF-κB and Akt1 [94, 95]. We observed that CAPE treatment significantly increased protein level of TRIB3, which may contribute to inhibitory roles of CAPE on NF-κB [17] and Akt1 (Figure 7).

Rb restricts cell cycle progression from the G1 phase to S phase [96–98]. Rb binds and inhibits transcription factors of the E2F family, which will result in G1 cell cycle arrest [96–98]. Phosphorylation of Rb is performed by cyclin D/Cdk4/Cdk6 and following by cyclin E/Cdk2. Phosphorylation of Rb blocks its binding to E2F and therefore allow the cells to progress from G1 phase to the S phase [96–98]. Rb remains phosphorylated throughout S, G2, and M phases. It is very interesting that we observed CAPE treated not only reduced the protein expression of E2F1 and phospho-Rb Ser807/811, but it also decreased the abundance of Rb. Previously, researchers observed that chemotherapeutic drug honokiol treatment induced G1 cell cycle arrest, growth inhibition, and induction of p21^Cip1^ and p53 in PC-3 and LNCaP cells [99]. Honokiol treatment significantly decreased in the levels of total and phosphorylated Rb, which correlated with the reduction of E2F1 transcriptional activity [99]. The authors concluded that honokiol treatment decreased protein levels of Cdk4, cyclin D1, and Rb via induction of proteasomal degradation [99]. We believe that CAPE also induced proteasomal degradation of Rb, cyclin D1, and Cdk4 in CRPC cells similar to honokiol as the response of CRPC cells to CAPE treatment is very similar to that of LNCaP cells being treated with honokiol.

**Figure 14:**
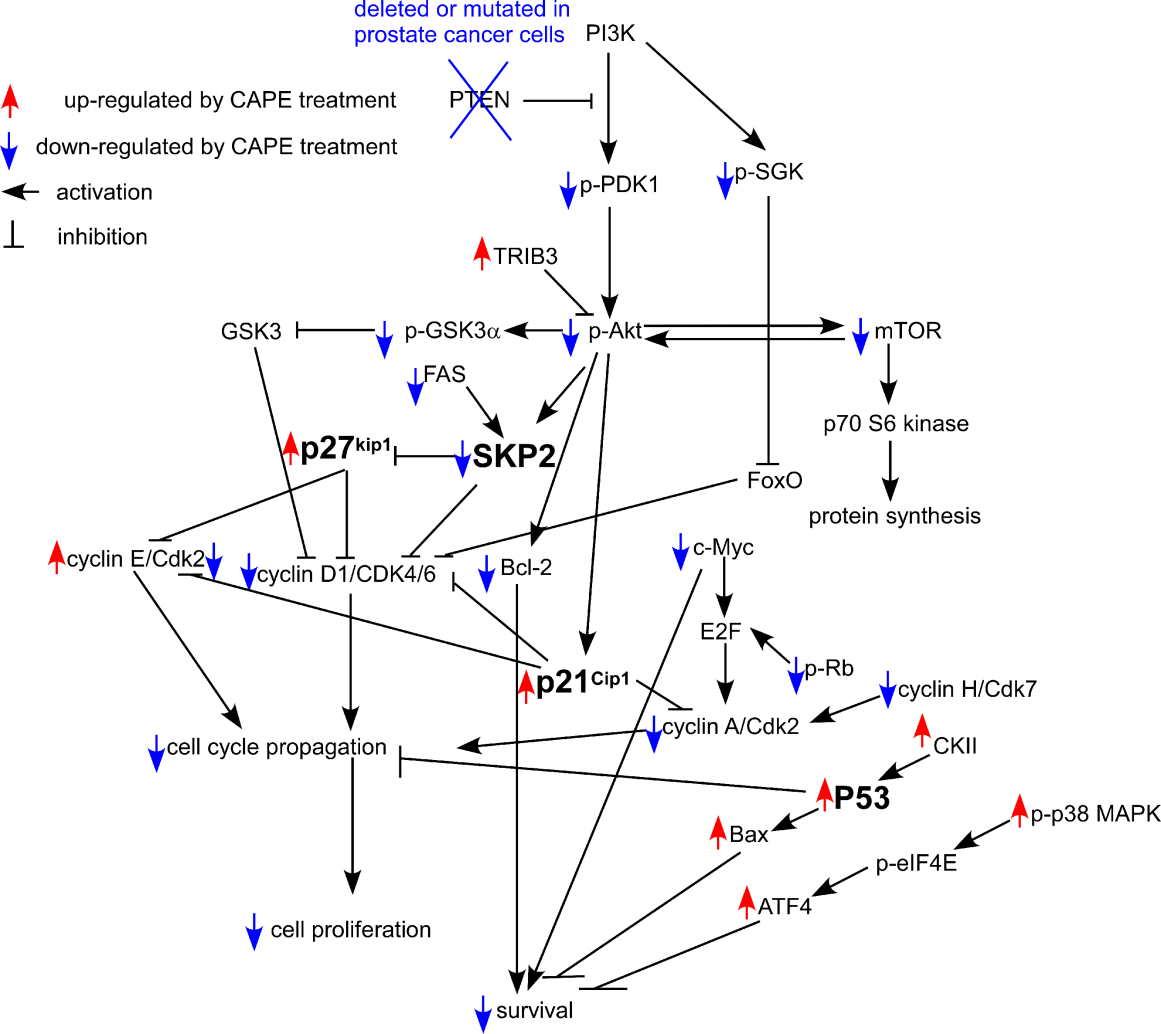
Putative model of anti-cancer effect of CAPE in human CRPC cells Protein abundance or activity being stimulated by CAPE treatment are labeled with red upward arrows, while those being suppressed by CAPE treatment are labeled with blue downward arrows. Arrows indicated activation of downstream signaling proteins, while bars means inhibition of downstream signaling proteins.

Our observation suggested that inhibition of Skp2 and induction p53, p21^Cip1^ and p27^Kip1^ was essential for growth inhibition promoted by CAPE treatment in CRPC cells. Not only did CAPE treatment suppressed Skp2 protein expression while increased protein abundance of p53, p21^Cip1^ and p27^Kip1^ (Figures 8, 9), but overexpression of Skp2 or siRNA knockdown of p53, p21^Cip1^ and p27^Kip1^ also partially blocked the suppressive effect of CAPE on CRPC cells (Figures 10, 11). We summarize all the signaling pathways being affected by CAPE treatment in CRPC cells in Figure 14.

We noticed that some Micro-Western Array revealed that CAPE treatment induced protein expression of Cdk2, cyclin D1, cyclin A, and SGK (Figure 5), while conventional Western blotting assay indicated that the abundance of these proteins was reduced by CAPE treatment (Figures 6, 7). According to our experience, there are approximately 5–20% inconsistency between MWA and conventional Western blot. The inconsistency is usually due to air bubbles affecting the image quality during protein transfer, weak signaling of certain MWA results, or cross-reactivity of certain antibodies. In Figure 10, the signal of Cdk2 was very weak, while the inconsistent results of SGK, cyclin A, and cyclin D1 may be affected by cross-reactivity of antibodies. We suggested that MWA can be used as a high-throughput screening tool similar to gene microarray while conventional Western blotting should be used to confirm the results as using RT-PCR for gene array confirmation.

The IC_50_ of CAPE treatment for AR-positive androgen-dependent LNCaP 104-S cells was 0.68 μM [19]. Compared to the parental LNCaP 104-S cells, 104-R1 cells is 28 fold more resistant to CAPE treatment. Under the culture condition, the protein abundance of Skp2, p21^Cip1^, p27^Kip1^, p53, total Akt, Akt1, Akt2, phospho-Akt S473, and phospho-Akt T308 are similar in 104-S and 104-R1 cells [21]. However, the protein level of Bcl-2 in 104-R1 cells is 65 fold higher than that in 104-S cells [21], and CAPE treatment caused very little inhibition on Bcl-2 level (Figure 12). Bcl-2 is an anti-apoptotic oncoprotein. Normal human prostate epithelial cells do not express the bcl-2 protein [100]. Up-regulation of Bcl-2 is necessary for the progression of LNCaP prostate cancer cells from an androgen-dependent to an androgen-independent growth stage [100, 101]. It is therefore very possible that high expression level of Bcl-2 proteins allows 104-R1 cells to be more resistant to CAPE treatment as compared to 104-S cells. In support of this hypothesis, co-treatment of Bcl-2 inhibitor ABT737 with CAPE showed synergistic suppressive effect (Figure 12), providing the rationale of using CAPE in combination with Bcl-2 for treatment of patients with CRPC.

We found that Tp53 gene level is lower in prostate tumors as compared to normal prostate epithelial tissue from Oncomine database analysis (Figure 13). Since CAPE treatment significantly increased abundance of p53 protein in CRPC cell lines, CAPE treatment may thus benefit patients with advanced prostate cancers.

## MATERIAL AND METHODS

### Chemicals

Caffeic acid phenethyl ester (CAPE) and LY294002 (PI3K inhibitor) were purchased from Sigma (St. Louis, MO, U.S.A.). CAPE was dissolved in ethanol for all cell experiments. RevertAid H Minus First Strand cDNA Synthesis Kit and SYBR Green/ROX qPCR Master Mix were purchased from Fermentas (Waltham, Massachusetts, U.S.A.). ABT737 (Bcl-2 inhibitor) was purchased from Santa Cruz (Santa Cruz, CA, U.S.A.). Matrigel was purchased from BD Bioscience (Franklin Lakes, NJ, U.S.A.).

### Cell culture

LNCaP 104-R1 cells were derived from parental androgen-dependent LNCaP 104-S cells, which were generated from LNCaP FGC clone (ATCC CRL-1740) as previously described [14, 102]. LNCaP 104-R1 cells were maintain in DMEM with 10% charcoal-stripped FBS (CS-FBS). PC-3, LNCaP C4–2, 22Rv1, and DU-145 cells were purchased from Bioresource Collection and Research Center (Hsinchu city, Taiwan). PC-3, LNCaP C4–2, 22Rv1, and DU-145 cells were maintained in DMEM (Gibco/Invitrogen, Carlsbad, CA, U.S.A.) supplemented with 10% fetal bovine serum (FBS; Atlas Biologicals, Fort Collins, CO, U.S.A.), penicillin (100 U/ml), and streptomycin (100 μg/ml) as previously described [21].

### Hoechst and DAPI staining miscroscopy

CRPC cells were seeded in 8 well chamber slide at a concentration of 1 × 10^4^ per well and was allowed to attach overnight. Cells were then treated with different concentration of CAPE 0, 2.5, 5, 10, 20 and 40 μM for 96 h. After 96 h, cells were rinsed twice with PBS and fixed with 4% formaldehyde for 15 minutes. Cells were then rinsed with PBS three times, each time for 5 min. After the rinsing, the fixed cells were Incubated in blocking buffer (5% BSA + 0.1% triton x-100 in PBS) overnight under at 4°C. After the blocking, cells were rinsed with PBS for three times, each time for 5 min. Finally, cells were stained with diluted 1:4000 Hoechst (stock solution 10 mg/mL) or DAPI (5 mg/mL) for 15 min and should be kept away from light exposure. Cells were then mounted into the slide and microscope images were captured at magnification of 100X.

### Cell proliferation assay

LNCaP 104-R1 cells, PC-3, LNCaP C4–2, 22Rv1, and DU-145 cells were seeded at a density of 3 × 10^3^ cells/well in 96-well plates with 100 μl DMEM medium containing 10% CS-FBS with increasing concentration of CAPE. Relative cell number was analyzed by measuring the DNA content of cell lysates with the fluorescent dye Hoechst 33258 (Sigma, St. Louis, MO, USA) as described previously [18, 19, 26, 62]. All readouts were normalized to the average of the control condition in each individual experiment. The experiment was repeated three times. Ten wells were used for each condition. The mean and standard deviation represented the average and standard deviation respectively of the results from all 30 wells in the three experiments.

### Cell viability assay

LNCaP 104-R1 cells were seeded at a density of 3 × 10^3^ cells per well in a 96-well plate (BD Bioscience). After 24 h, the cells were treated with increasing concentrations of CAPE for 96 h. Cell viability was assessed by an MTT (3,4,5-dimethylthiazol-2-yl)-2–5-diphenyltetrazolium bromide) assay [103]. The amount of formazan was determined by measuring the absorbance at 560 nm using an Tecan GENios™ plate reader (Tecan group Ltd, Männedorf, Switzerland) [103]. All results were normalized to the average of the control condition in each individual experiment. All experiments were repeated three times. Each time ten wells were utilized for each condition. The mean and standard deviation represented the results from all 30 wells in the three experiments.

### Soft agar colony formation assay

We suspended 8,000 LNCaP 104-R1 cells in 0.3% low melting agarose (Lonza) with 10% CS-FBS in DMEM medium and then layered on top of 3 ml of 0.5% low melting agarose plus 10% CS-FBS in DMEM medium in 6 cm dishes. Cells were allowed to grow at 37°C with 5% CO_2_ for 14 days. The plates were stained with 0.005% crystal violet in 30% ethanol for 6 h.

### Flow cytometric analysis

After 96 h of culture in the presence of different concentrations of CAPE, cells were processed as previously described [21, 26, 62]. Cell cycle profiles of LNCaP 104-R1, DU-145, 22Rv1, and LNCaP C4–2 cells were determined by flow cytometric analysis using a BD Facscan flow cytometer (BD Biosciences, San Jose, CA). Data was analyzed using ModFit LT software (Verity Software House, Topsham, ME) as described [21, 26, 62].

### Western blotting analysis

Cells were lysed in SDS lysis buffer (240 mM Tris-acetate, 1% SDS, 1% glycerol, 5 mM EDTA pH 8.0) with DTT, protease inhibitors, and a cocktail of phosphatase inhibitors. Anti-rabbit and anti-mouse IgG secondary antibodies were from Invitrogen (Carlsbad, CA, U.S.A.) and LI-COR BioSciences (Lincoln, Nebraska). Akt, phospho-Akt Ser473, phospho-Akt Thr308, Rb, phospho-Rb (S807/811), cyclin D1, cyclin E, Cdk2, phospho-Cdk2 Thr160, p38 MAPK, GSK3α, phospho-GSK3α Ser21, GSK3β, phospho-GSK3β Ser9, mTOR, phospho-mTOR Ser2481, phospho-CREB Ser133, phospho-ATM S1981, phospho-ATR S428, and Bax antibodies were purchased from Cell Signaling Technology (Danvers, MA, U.S.A.). Skp2, ATF-4, p27^Kip1^ and p21^waf1/cip1^ antibodies were from purchased Santa Cruz. P70S6kinase, phospho-P70S6K Thr241/Ser424, p53, phospho-p53 Ser6, phospho-p53 Ser33, phospho-p53 Ser46, phospho-p53 Ser392, fatty acid synthase (FAS), androgen receptor (AR), c-Myc, phospho-CDK2 Thr14, phospho-CDK2 Tyr15, CDK4, SGK, p90 RSK1, phospho-p90 RSK1 Ser380, PDK1, phospho-PDK1 Ser241, Casein Kinase I, Casein Kinase IIα, Casein Kinase IIβ, CREB, and α-tubulin antibodies were purchased from Epitomics (Burlingame, CA, USA). Bcl-2 was purchased from BD BioSciences (San Jose, CA, USA). Akt2 and β-actin were purchased from Novus (Littleton, CO, U.S.A.). Bad, phospho-p38 Thr180/Tyr182, E2F-1, Cyclin A, Akt1, phospho-SGK Ser255/Thr256, and phospho-mTOR Ser2448 antibodies were from Millipore (Billerica, MA, U.S.A.). TRIB3, Cdk7, and Cyclin H antibodies were from Abnova (Taipei, Taiwan). Chk1 and Chk2 antibodies were purchased from Abcam (Cambridge Science Park, Cambridge, UK). Blots were scanned and quantified using a LI-COR Odyssey near-infrared imaging system. Horseradish peroxidase-conjugated anti-rabbit and anti-mouse IgG secondary antibodies were purchased from Santa Cruz. α-tubulin and β-actin were used as loading controls.

### Quantitative real-time polymerase chain reaction (qRT-PCR)

Cell RNA was extracted from LNCaP 104-R1 cells treated with ethanol (control), 10 μM, 20 μM or 40 μM CAPE for 48 h by RNeasy Mini kit. Cell pellet was lysed by Buffer RLT. The mRNA expression of Akt1, Akt2, cyclin D, c-Myc and Skp2 p45 were assayed using SYBR Green real-time PCR arrays. GAPDH was assayed as RNA content loading control in each array. The transcript level of selected genes was analyzed using the RT^2^ Profiler PCR Array Data Analysis website (http://www.sabiosciences.com/pcr/arrayanalysis.php) and normalized to GAPDH levels [26].

### Micro-western arrays

LNCaP 104-R1 cells were treated with 0, 10, 20, or 40 μM CAPE for 96 h. Three biological replicates of cells were lysed in SDS lysis buffer (240 mM Tris-acetate, 1% SDS, 1% glycerol, 5 mM EDTA pH 8.0) with DTT, protease inhibitors, and a cocktail of phosphatase inhibitors. Micro-Western Arrays were performed to measure protein expression and phosphorylation status modification as previously described [22, 104]: Gel fabrication. Glass casting plates (one measuring 14 × 27 cm, the other measuring 14 × 28 cm) were sprayed with BlueSlick (Serva) and wiped thoroughly. Rubber spacers were placed on three sides of the inner coated sides of the glass plate. One rectangle of Netfix (Serva) was placed on the glass plate on top of the spacers. The second glass plate was placed on top with the coated surface facing down. Twelve clamps were placed around the three gasketed edges of the sandwich. Gel reagents: For 10% acrylamide gel, we gently mixed 6 ml of 5× gel buffer (1.2 M Tris-Acetate, adjust pH of Tris-base (Sigma) with acetic acid (Fisher) to 6.9), 10 ml acrylamide (Ultrapure) 3.8% crosslinker (Fisher), 7.5 ml ddH_2_O, 6 ml Neat glycerol, 300 μl 10% w/v SDS, 150 μl 10% APS, and 12 μl Ultrapure TEMED (Invitrogen). This is enough for 2 gels. A 30 ml syringe with a 19 gauge needle was used to inject the gel mixtures. The sandwich was laid horizontally for an hour. The gel was then removed from the bottom glass plate. Microarraying: The lysates were spotted using a noncontact microarrayer (GeSiM Nanoplotter 2.1E) with active humidification. Z-height measurements were taken before the print of each gel. The printing performance of each tip was validated with a stroboscope before beginning each microarray print. If printing was inconsistent, 200 μl of 50% methanol–50% HCl was loaded into the tip and dispensed three times using manual mode. The tips were washed for 60 s using the wash/dry cycle and rechecked with the stroboscope. MWAs were printed onto either one or two gels per array run. Tip dispense height was held at 1.5 mm above the gel surface while printing. Samples were placed with the ladder in well A1 of a 384 well plate and samples consecutively in A2–A7. Software NP2.15.46 was used. The TransferTipMultiSim04H9 (GeSiM) was run using the transfer text and the workplate definition file provided in Supplementary Note 3 as reported previously [22, 104]. Odyssey protein ladder (LI-COR) was printed in lane 1 at a 1:2 dilution in lysis buffer. After print completion, the gel was subsequently rehydrated for 5 min in the rehydration buffer described above with gentle agitation. After rehydration, the gel was placed onto the multiphor (GE Healthcare) for horizontal dry electrophoresis. Horizontal dry electrophoresis: Samples were separated by size using a multiphor (GE Healthcare). The power supply was set at 350 V, 30 W and unlimited amps. The lowest-molecular-weight ladder bands migrated about 9 mm (the length of one well of a 96-well plate) in 12 min. Transfer: After electrophoresis, the gel was placed protein side down onto nitrocellulose (Bio-Rad) premoistened in transfer buffer (25 mM Tris base, 0.2 M glycine (Fisher), 20% methanol (Sigma), pH 8.5). Filter paper was placed on either side of the nitrocellulose and gel, and was clamped in a transfer box cartridge. Bubbles were pressed out with a roller. The gels were transferred either at 0.8 A for 60 min or 0.15 A overnight at 4°C in a Criterion transfer box (Bio-Rad) with plate electrode. Blotting: Nitrocellulose was removed from the transfer apparatus and washed for 5 min in TBS (without Tween 20) to remove methanol. The blots were blocked for 1 h in Odyssey blocking buffer (LI-COR). The blot was aligned on the gasket by placing the visible ladder on the vertical lines and centering the ladder between the horizontal lines. The gasket was clamped into the 96-well isolation device, and primary antibodies were pipetted into the appropriate wells, making sure that the membrane remained wet during the process. The primary antibody was diluted in pure Odyssey blocking buffer (without Tween 20) overnight. We added 150 μl of diluted antibody per well. After incubation, the wells were washed four times with 200 μl of TBST per well using a multichannel pipettor. Goat anti-rabbit Alexa Fluor 680–conjugated secondary (Invitrogen), goat anti-rabbit and goat anti-mouse IR800-conjugated secondary antibodies (1:5,000) (LI-COR) were diluted in 20% Odyssey blocking buffer, 80% TBS (without Tween 20). We added 150 μl of the diluted secondary antibody to the appropriate well. After incubation for an hour, the blot was washed three times with 200 μl TBST while clamped in the gasketing device (Arrayit). The blot was then removed from the gasket, placed in a box top and washed for an additional 5 min in TBST. For the fifth wash, TBS without Tween 20 was used, washing for 5 min. The membrane was completely dried using pressurized air and scanned using the LI-COR Odyssey imager at 24 μm resolution and high quality (using laser intensity 1.0 on the 700 nm channel, and using laser intensity 2.0 on the 800 channel) settings. Analysis: Scanned images were saved for analysis as 16-bit tiff files. Genepix 8.0 (Molecular Devices) was used to record the mean by drawing an equally sized circle around the appropriately sized band for each sample. Appropriate size was defined as within 10 kDa of the size as defined by the antibody product sheet as measured in comparison to the LI-COR ladder bands. All bands within this region that were visible were recorded. Bands outside this region were noted but the intensities were not recorded or analyzed. The background fluorescence was recorded by placing an equal sized circle in the blank space to the left of the first sample (not covering sample or ladder space) and the minimum value of this circle was recorded. Net intensity was calculated by subtracting each sample intensity from the background. To normalize sample concentration, the net intensities were divided by a simple mean of the net intensities for GAPDH, α-tubulin and β-actin calculated separately for each array print. Fold change was calculated as the ratio of each normalized net intensity to the net intensity at the 0 min time point, minus one.

### Protein overexpression

Ectopic expression of Akt1 and c-Myc was achieved by infecting LNCaP 104-R1 cells with pSRα or pBabe retroviruses carrying the cDNA of the indicated proteins, respectively. Antibiotic-resistant (G418 and puromycin) colonies were expanded and screened for increased target protein expression by western blot analysis. For Skp2 overexpression, LNCaP 104-R1 cells were infected with pMV7 retrovirus containing Skp2 inserts that was generated in ΦNX-Ampho packaging cells using procedures described previously [14]. The ΦNX-Ampho packaging cell line was provided by Garry Nolan of Stanford University. Stably infected cells were selected by G418. Cells infected with retrovirus carrying empty vectors were used as controls.

### siRNA knockdown of p53, p27^Kip1^ and p21^Cip1^

Human p53, p27^Kip1^, p21^Cip1^ antisense and randomly scrambled sequence control were purchased from GE Healthcare (Little Chalfont, United Kingdom). The transfection procedure was performed using lipofectamine RNAiMAX (Invitrogen, Carlsbad, CA, U.S.A.) according to the manufacturer's recommended protocol. 40 nM RNA were used for scramble, p53, p27^Kip1^ and p21^Cip1^ knockdown.

### Cellular senescence assay

5 × 10^4^ LNCaP 104R1 cells were seeded in each well of 6-well plate with DMEM containing 10% CS-FBS. 24 h after plating, cells were treated with increasing concentrations of CAPE. After an additional 96 h, cells were washed with PBS and added 1 mL of Fixing Solution per well (Millpore, Billerica, MA, U.S.A.). Following 15 min room temperature incubation, the cells were washed twice with PBS and added 2 mL of senescence-associated betagalactosidase (SA-β-gal) Detection Solution. After incubating at 37°C without CO_2_ overnight, the cells were washed twice with PBS and photo image of the cells were taken with phase contrast microscopy or light microscopy.

### Xenografts in athymic mice

Experiments involving mice were approved by National Health Research Institutes Institutional Animal Care and Use Committee (NHRI-IACUC-101115-A). The study was carried out in strict accordance with the recommendations in the Guide for the Care and Use of Laboratory Animals of the National Institutes of Health. Male Balb/c nu/nu mice purchased from National Laboratory Animal Center (Taipei city, Taiwan) at age 6–8 weeks of age were injected subcutaneously in both flanks with 5 × 10^5^ LNCaP 104-R1 cells suspended in 0.5 ml of Matrigel (BD Bioscience, Franklin Lakes, NJ, USA) and were injected subcutaneously into athymic mice to form tumors. After 14 weeks, the average tumor volume exceeded 150 mm^3^. The mice were then separated into control group and CAPE treatment group. Control group contained 6 mice and 8 tumors, while CAPE treatment group contained 6 mice and 9 tumors. CAPE (10 mg/kg/day in sesame oil) or vehicle (sesame oil) was administered by gavage starting from 14th week after cancer cell injection. Tumor volume and body weight of mice carrying 104-R1 xenografts was measured weekly using calipers and volume was calculated using the formula volume = length × width × height × 0.52 [62, 105–107]. Tumor samples for Western blotting assay analysis were prepared from tissue homogenized in 2× Laemmli buffer as previously described [14, 106].

### Combined treatment of CAPE with PI3K inhibitor LY294002 or BCl-2 inhibitor ABT737

Proliferation of LNCaP 104-R1 cells treated with combination of CAPE with LY294002 or CAPE with ABT737 was determined by 96-well proliferation assay. The relative cell number under combined treatment was compared to that being treated with only CAPE, LY294002, or ABT737 alone. The effect of the combined treatment was determined by the ratio of expected cell number/observed cell number. For example, treatment of 104-R1 cells with 5 μM of ABT737 decreased the cell number to 69.2% and treatment with 104-R1 cells with 5 μM CAPE alone decreased the cell number to 75.6%. The expected cell number of treatment combining 5 μM of ABT737 with 5 μM CAPE was 0.692 × 0.756 = 52.3%. The actual observed cell number is 47.0%. The ratio of expected cell number/observed cell number is 0.523/0.470 = 1.1. Ratio larger than one represents synergy of growth inhibition as the combined treatment of two drugs suppressed more cells than either drug alone. If the observed cell number is less than the cell number being treated with any one of the drug alone, this indicates additive suppressive effect of the combination treatment of the two drugs. For example, treatment of 104-R1 cells with 2.5 μM of LY294002 decreased the cell number to 91.2%, while treatment with 104-R1 cells with 40 μM CAPE decreased the cell number to 14.5%. The combination of 2.5 μM of LY294002 with 40 μM CAPE decreased the cell number to 13.1%, then we called the combination of 2.5 μM of LY294002 with 40 μM CAPE exhibited additive suppressive effects on LNCaP 104-R1 cells.

### Public domain data

Expression profile of Tp53 gene from Singh prostate datasets was detected by reporter probe 1974_s_at with Human Genome U95A-Av2 Array [108], which contains 50 normal prostate gland samples and 52 prostate carcinoma samples. Expression profile of Tp53 gene from Tomlins prostate datasets was detected by reporter probe IMAGE:24415 [109], which contains 21 normal prostate gland epithelial samples, 4 BPH (benign prostatic hyperplasia), 12 PIN (prostatic intraepithelial neoplasia), and 30 prostate carcinoma samples. Data were downloaded from Oncomine (http://www.oncomine.com) without further processing.

### Data analysis

Data are presented as the mean +/– SD of at least three experiments or are representative of experiments repeated at least three times. Student's *t*-test (two-tailed, unpaired) was used to evaluate the statistical significance of results from the proliferation assay experiments. A Microsoft Excel add-in program ED50V10 was used for calculating half maximal effective concentration (EC50).

## CONCLUSION

Our finding suggested that treatment with CAPE caused cell cycle arrest and growth inhibition in CRPC cells both *in vitro* and *in vivo* via inhibition of Skp2 and induction p53, p21^Cip1^ and p27^Kip1^. We believe that CAPE treatment may be a novel and useful therapy for patient with CRPC.

## SUPPLEMENTARY FIGURES


