# Supplementary material for: Caffeic acid phenethyl ester induced cell cycle arrest and growth inhibition in androgen-independent prostate cancer cells via regulation of Skp2, p53, p21Cip1 and p27Kip1
Source: Oncotarget. 2015 Feb 16;6(9):6684–707. doi: 10.18632/oncotarget.3246 (PMC4466643; doi:10.18632/oncotarget.3246)
Supplement: Supplementary file 1 [file oncotarget-06-6684-s001.pdf]

SUPPLEMENTARY FIGURES

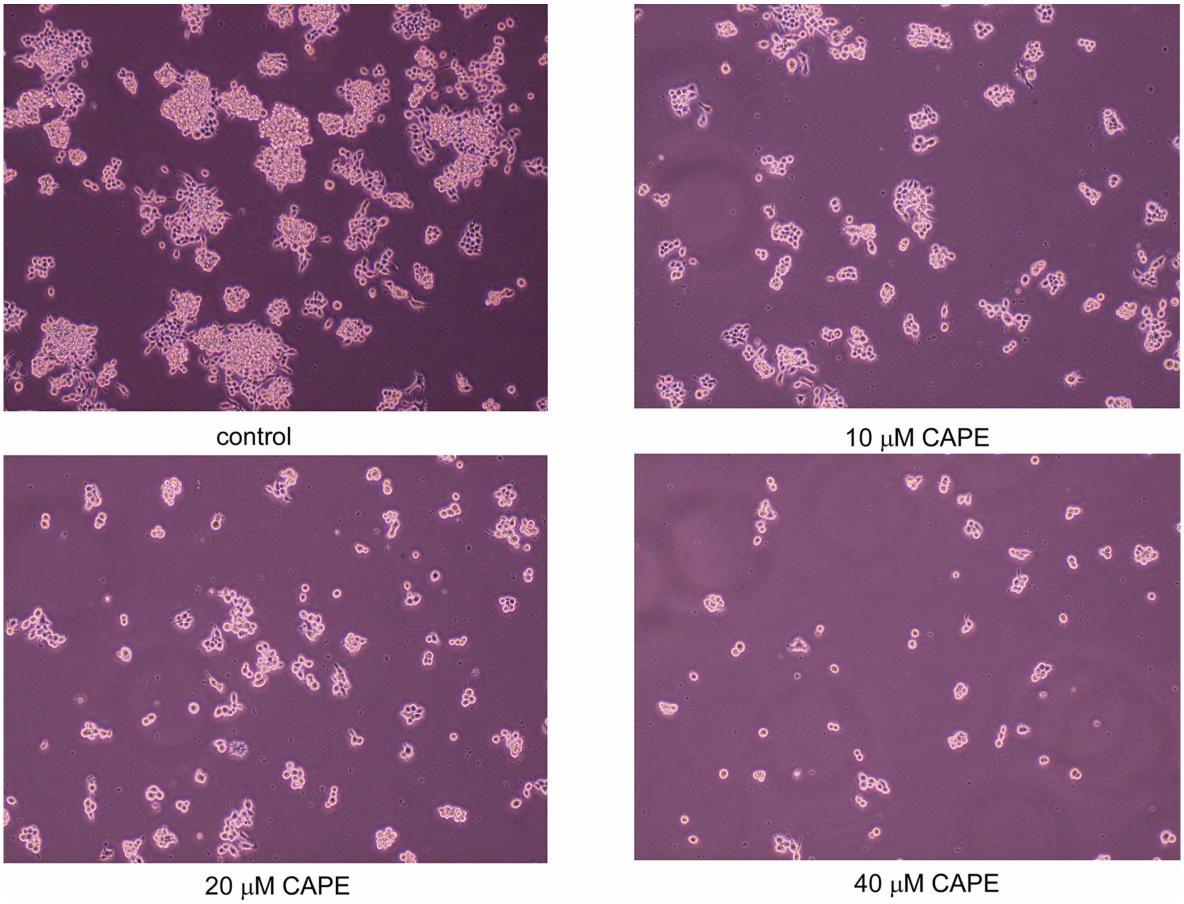

Supplementary Figure 1: CAPE treatment for 96 h reduced cell number of LNCaP 104-R1 cells. Images of LNCaP 104-R1 cells being treated with increasing concentrations of CAPE for 96 h were observed using light microscope with magnification of 100X.

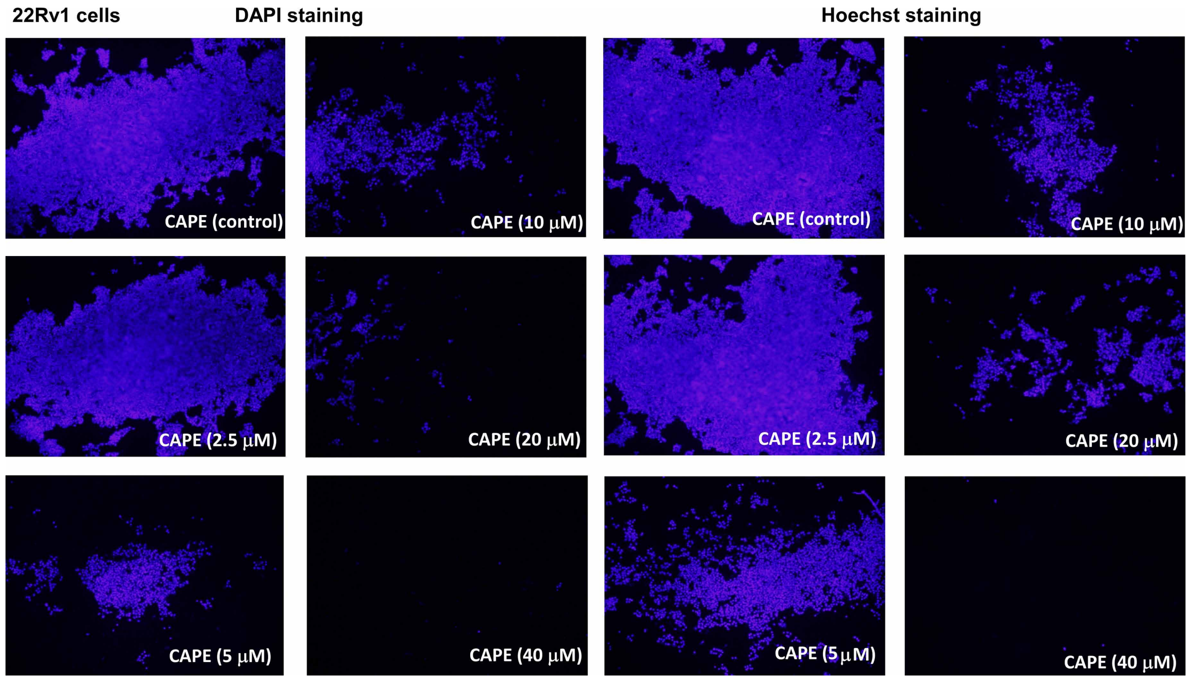

Supplementary Figure 2: CAPE treatment for 96 h reduced cell proliferation of 22Rv1 cells. DAPI staining and Hoechst dye-staining of 22Rv1 cells being treated with increasing concentrations of CAPE for 96 h was used to monitor cell proliferation of 22Rv1 cells using fluorescent microscope with magnification of 100X.

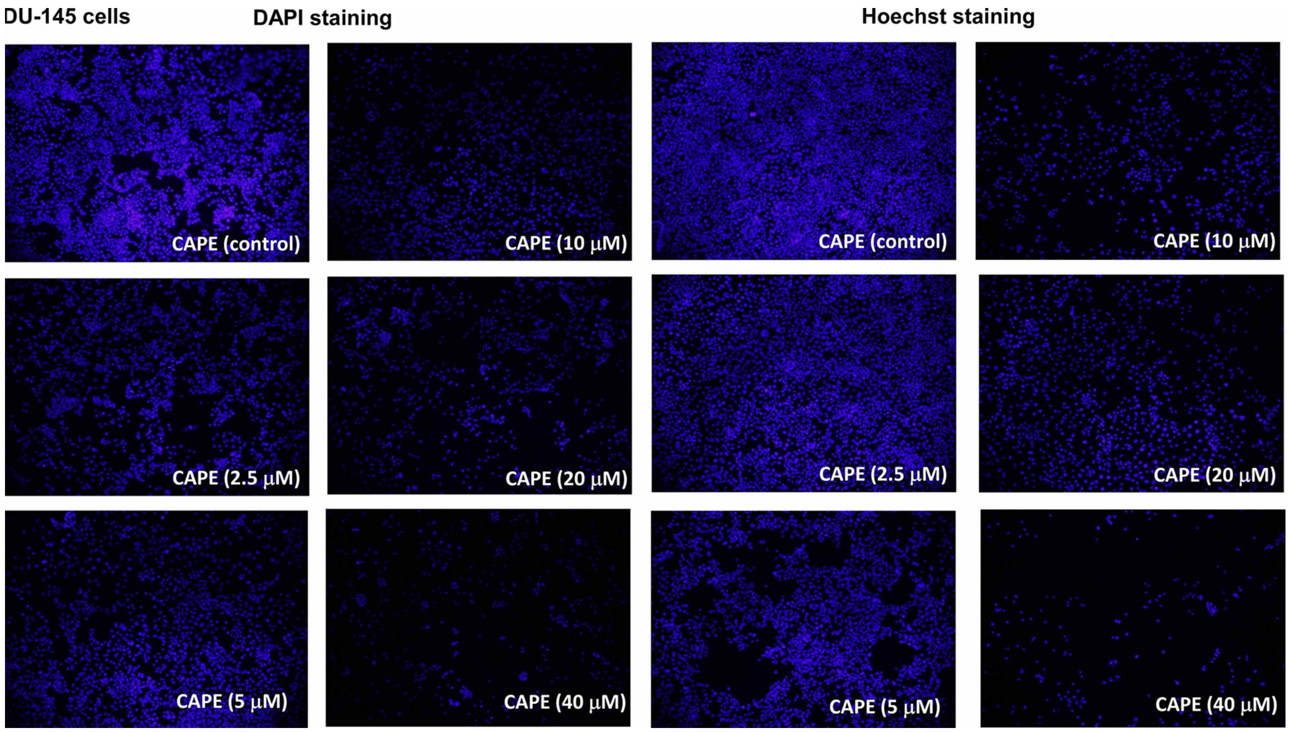

Supplementary Figure 3: CAPE treatment for 96 h reduced cell proliferation of DU-145 cells. DAPI staining and Hoechst dye-staining of DU-145 cells being treated with increasing concentrations of CAPE for 96 h was used to monitor cell proliferation of DU-145 cells using fluorescent microscope with magnification of 100X.

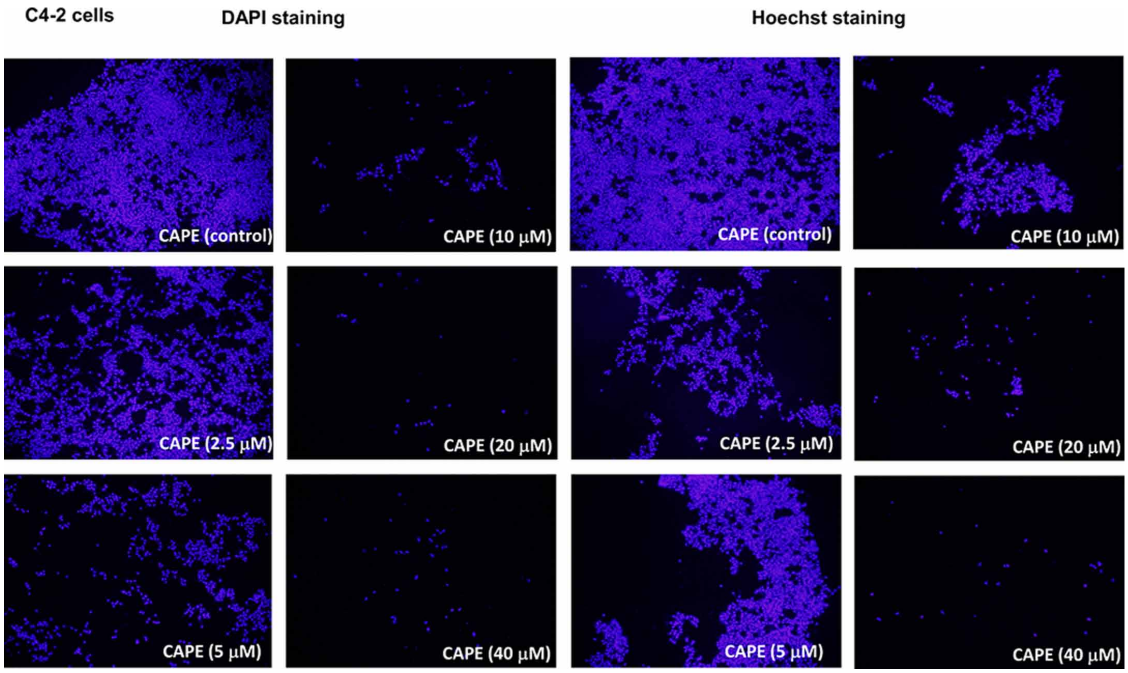

Supplementary Figure 4: CAPE treatment for 96 h reduced cell proliferation of C4–2 cells. DAPI staining and Hoechst dye-staining of C4–2 cells being treated with increasing concentrations of CAPE for 96 h was used to monitor cell proliferation of C4–2 cells using fluorescent microscope with magnification of 100X.

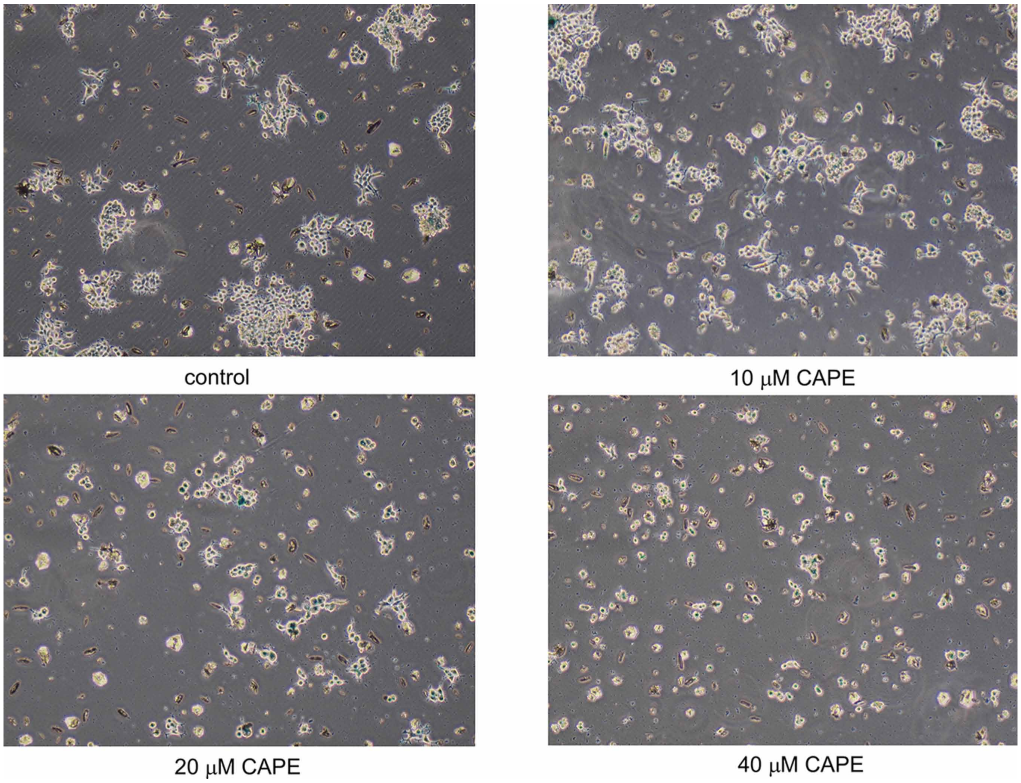

Supplementary Figure 5: CAPE treatment induced moderate β-galactosidase staining in LNCaP 104-R1 cells as determined by β-galactosidase staining. LNCaP 104-R1 cells were treated with 0, 10, 20, 40 μM CAPE for 96 h. Expression of β-galactosidase was stained to determine if CAPE treatment causes cellular senescence and observed using light microscope with magnification of 100X.
